# NIR-triggered programmable nanomotor with H_2_S and NO generation for cascading oncotherapy by three-pronged reinforcing ICD

**DOI:** 10.1016/j.mtbio.2025.101540

**Published:** 2025-02-03

**Authors:** Jinlong Zhang, Quan Jing, Longlong Yuan, Xianhui Zhou, Duolong Di, Jinyao Li, Dong Pei, Zhongxiong Fan, Jun Hai

**Affiliations:** a CAS Key Laboratory of Chemistry of Northwestern Plant Resources and Key Laboratory of Natural Medicine of Gansu Province, Lanzhou Institute of Chemical Physics, Chinese Academy of Sciences, Lanzhou, 730000, Gansu, China; b School of Pharmaceutical Sciences, Institute of Materia Medica, Xinjiang University, Urumqi, 830017, China; c University of Chinese Academy of Sciences, Beijing, 100049, China; d Department of Cardiac Pacing and Electrophysiology, The First Affiliated Hospital of Xinjiang Medical University, Urumqi, 830054, China; e Xinjiang Key Laboratory of Biological Resources and Genetic Engineering, College of Life Science and Technology, Xinjiang University, Urumqi, 830017, China

**Keywords:** Gas signal molecule, Nanomotor, Mild photothermal therapy, Immunogenic cell death, Oncortherapy

## Abstract

Gas therapy (GT) and/or phototherapy have been recently employed as immunogenic cell death (ICD) agents for activating immunotherapy, whereas the effective activation of sufficient immune responses remains an enormous challenge in such single therapeutic modality. In this study, a near-infrared (NIR)-triggered programmable nanomotor with hydrogen sulfide (H_2_S) and nitric oxide (NO) generation is well designed to achieve oncotherapy by cascading mild photothermal, gas, and reactive oxygen species (ROS)-reinforced immunogenic cell death. In brief, a gas signal molecule donor NOSH with H_2_S and NO capable of on-demand H_2_S and NO release was synthesized and then loaded into hollow mesoporous copper sulfide nanoparticles (termed as HCuSNPs) with an inherent NIR absorption and surface modification activity to obtain the programmable nanomotor (termed as NOSH@PEG-HCuSNPs). In particular, NOSH@PEG-HCuSNPs can effectively achieve the simultaneous spatiotemporal co-delivery of NOSH and HCuSNPs, thereby exerting the synergistic effects of GT and mild photothermal therapy (mPTT). It is worth noting that the anti-tumor response of mPTT is effectively enhanced by GT by disrupting the mitochondrial respiratory chain, inhibiting ATP production, and promoting tumor cell apoptosis. One by one, a large number of peroxynitrite anion (ONOO^−^) radicals are generated by the interactions of ROS from mPTT and NO from NOSH. Meanwhile, the unique protective mechanism of H_2_S is utilized to induce tumor thermal ablation by reducing the overexpression of heat shock protein 90 (HSP 90) and minimize the unnecessary damage toward normal tissues. Finally, ICD is markedly augmented by the cascading effects of mPTT, ONOO⁻radicals, and H_2_S. Concurrently, the immunosuppressive tumor microenvironment is reprogrammed, effectively inhibiting distant tumor tissues and preventing metastasis and tumor recurrence. Taken together, this study provides a new perspective for innovation in the field of oncotherapy.

## Graphical abstract

**Figure:**
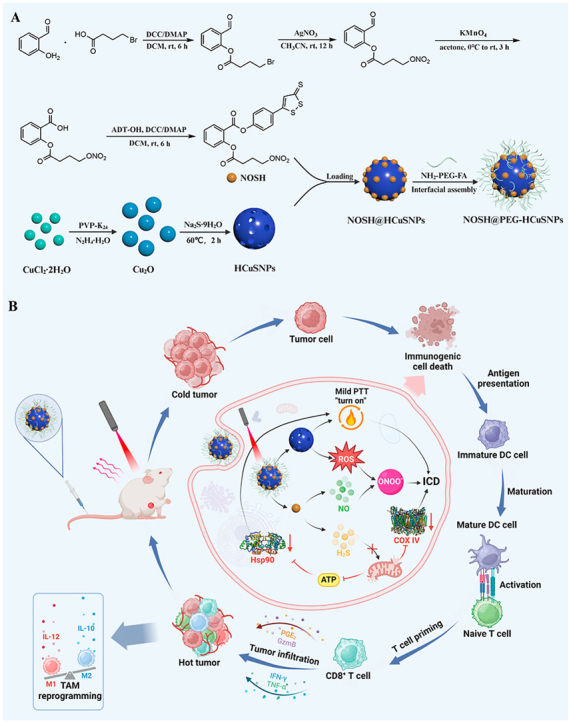

## 1 Introduction

Gas signaling molecules are important transmitters in living organisms. Such molecules can precisely bind to the corresponding receptors and trigger specific biological effects to transmit information or induce specific physiological reactions, and thus play an important regulatory role [1,2]. In recent years, gas therapy (GT) based on *in situ* generation of gaseous signaling molecules such as nitric oxide (NO), carbon monoxide (CO), hydrogen sulfide (H_2_S), and hydrogen (H_2_) has attracted great interest in oncotherapy due to its excellent therapeutic effect and biosafety [3]. Typically, H_2_S can activate intracellular acidification, induce cell cycle arrest, inhibit promoting cell survival pathways, and ultimately result in tumor apoptosis [4]. In addition, H_2_S can disrupt mitochondrial homeostasis by reducing the activation of cytochrome *c* oxidase (COX IV), interfere with the respiratory chain, and inhibit cellular energy supply to hinder tumor proliferation [5]. Notably, H_2_S also has a cytoprotective effect, which can be conducive to repairing overactive inflammation, accelerating wound healing, maintaining intracellular redox homeostasis [6]. Therefore, H_2_S as a natural repair agent can reduce the damage of invasive anti-cancer methods. A large number of studies have found that heat shock protein 90 (HSP 90) as a key protein can induce cell heat resistance [7], and its expression is positively correlated with ATP levels [8]. Besides, H_2_S-induced depletion of intracellular ATP pools is considered to block the production of HSP 90, thereby reversing resistance to invasive oncotherapy [9]. It is also found that NO can exert its tumor-killing effect by inducing mitochondrial/DNA damage, blocking DNA synthesis and repair, and inhibiting cell respiration [10]. In addition, NO as a natural regulator can also change the immunosuppressive tumor microenvironment. On the one hand, NO itself can overcome resistance resulted from oncotherapy by activating immune cells, inhibiting immunosuppressive factors, promoting immune cell infiltration, and promoting tumor vascular normalization [11,12]. On the other hand, the latest studies have confirmed that NO can react with reactive oxygen species (ROS) generated during various phototherapy processes to generate peroxynitrite anion (ONOO^−^), which is a reactive nitrogen species (RNS) that has a stronger inhibitory effect on tumor growth than traditional •OH and •O_2_^−^ [13,14]. What's more, the generation of RNS can effectively reverse the immunosuppressive tumor microenvironment by regulating the polarization of macrophages to an anti-tumor state, thereby enhancing the therapeutic efficacy of various tumors [15,16]. Therefore, GT involving multiple gas signaling molecules may become a promising direct therapy and indirect auxiliary anti-tumor methods.

Photothermal therapy (PTT) is as an emerging and promising strategy for various diseases can convert light energy into heat energy to directly kill tumor cells by thermal ablation of tumor tissues [17]. In order to achieve the tumor ablation, the pathological site is generally heated to more than 50 °C by the characteristics of photothermal agents. Although the increase in local temperature is effective for tumor elimination, it will inevitably lead to inevitable damage toward adjacent normal tissues through thermal diffusion [18]. In addition, skin thermal swelling induced by photothermal hyperthermia also may induce long-term inflammation and hyperimmunogenicity, thereby inducing serious side effects [19]. In contrast with conventional PTT, mild photothermal therapy (mPTT) (the operating temperature <45 °C) can significantly reduce the damage risk toward normal skin [20]. However, due to the limited radiation efficiency of the therapeutic laser used in mPTT, the blood flow in the therapeutic region rapidly dissipates heat. Hence, mPPT are difficult to kill tumor cells at the tumor edge and around blood vessels beyond the laser penetration limit, which will result in this fact that tumor are not thoroughly cleared and further developed into invasive tumor to continue proliferation and recurrence of tumor [21]. In addition, some authoritative studies have revealed that the immunosuppressive microenvironment of tumor can be beneficial for tumor cells to escape the attack of the immune system and promote the growth, invasion, and metastasis of tumor cells [22]. Although mPTT can reshape the tumor immunosuppressive microenvironment by inducing immunogenic cell death (ICD) during treatment, the interaction between the tumor microenvironment and the immune response after treatment strengthens the tumor's immunosuppressive state. As a result, the tumor areas that have not been completely cleared bypass the host's immune response, thereby increasing the risk of mPTT recurrence [9]. Therefore, how to explore some effective methods to regulate the tumor immunosuppressive microenvironment, break through the tumor immune biological barrier, reduce the risk of metastasis and recurrence, and thus improve the therapeutic effect and maximize the clinical potential of mPTT. For example, how to skillfully combine mPPT with other therapeutic methods (photodynamic therapy (PDT), chemotherapy, chemodynamic therapy (CDT), ferroptosis) is expected to become a promising oncotherapy strategy.

In recent years, the combination strategy of GT and mPTT has attracted widespread attention in oncotherapy [23]. Regrettably, existing treatment methods are simply a complex superposition of photothermal therapy and other treatments, rather than a simple treatment strategy that cascades the synergy between photothermal therapy and various treatment methods [24]. Therefore, to further improve the potential application value of mPTT with a simple system as much as possible and achieve ideal therapeutic effects and harmless therapeutic processes, how to explore mPTT-based other new strategies and develop nanoplatforms is urgent It should be pointed out that the long-term exposure to H_2_S or NO does not impair the viability of normal cells, which provides feasibility for the synergistic targeted oncotherapy with H_2_S and NO [25,26]. Although the importance of H_2_S and NO has becoming increasingly apparent in anti-tumor occurrence on account of their unstable chemical properties, it is difficult to effectively deliver therapeutic levels of H_2_S and NO to tumor tissues [6,27]. On the one hand, this delivery difficulty derived from the obvious dose-dependent activity of gas signal molecules. In sharp contrast to high concentrations, low levels of H_2_S and NO stimulate tumor occurrence by accelerating the cell cycle, promoting proliferation, and inhibiting apoptosis [28,29]. On the other hand, it is a great challenge to ensure that the two gas signal molecules are simultaneously targeted and delivered to the tumor site [30]. Therefore, a gas donor that can release H_2_S and NO continuously and simultaneously in sufficient quantity is introduced into the photothermal nanoplatform to reverse the immunosuppressive microenvironment and achieve precise coordination of GT and mPTT. Using a single regulation to achieve efficient anti-cancer effects has very important application prospects.

In this paper, we first designed and synthesized a gas signal molecule donor NOSH that releases H_2_S and NO on demand to enhance the near-infrared-induced mPTT effect [31]. Subsequently, large-cavity mesoporous copper sulfide nanoparticles HCuSNPs with fixed near-infrared (NIR) absorption and surface modification activity were constructed, which not only exhibited excellent photothermal conversion efficiency but were also considered to be one of the promising biodegradable and biocompatible photothermal conversion agents [32]. On this basis, we loaded the gas donor NOSH onto the photothermal agent HCuSNPs and modified it with polyethylene glycol (PEG) derivatives that can improve its biocompatibility and tumor targeting [33],constructing a programmable nanomotor NOSH@PEG-HCuSNPs. This nanomotor can not only achieve the synchronous delivery of NOSH and HCuSNPs, but also play an effective synergistic role between GT and mPTT. NOSH@PEG-HCuSNPs can induce additional mitochondrial dysfunction during tumor thermal ablation under near-infrared laser irradiation, thereby enhancing tumor cell apoptosis. In addition, by utilizing the unique protective mechanism of H_2_S, the overexpression of HSP 90 during tumor thermal ablation was reduced, which greatly reduced the unnecessary damage to normal tissues during laser irradiation. A large number of ONOO^−^ free radicals with strong tumor inhibition were generated by the interaction between ROS in mPTT and NO in NOSH. Therefore, the cascade reaction of mPPT, ONOO^−^ free radicals and H_2_S enhanced tumor ICD, reversed the tumor immune microenvironment, and effectively prevented tumor recurrence and metastasis. This strategy confirmed the feasibility of non-invasive mPTT to cure tumors and showed great application potential.

## 2 Materials and methods

### 2.1 Reagents and instruments

Unless otherwise specified, the reagents used in this experiment are analytically pure and do not require further purification. All reagents were purchased from legal commercial channels. Copper (II) chloride dihydrate (CuCl_2_·2H_2_O) and Sodium sulfide nonahydrate (Na_2_S·9H_2_O) were obtained from Chengdu Kelong Chemical Co., Ltd. Poly (vinylpyrrolidone) (PVP K40, Mw = 40,000) was purchased from Sigma-Aldrich. Hydrazine hydrate aqueous solution (N_2_H_4_·H_2_O) was provided by Shanghai Zhongqin Chemical Reagent Co., Ltd. L-Arg, 1-(3-Dimethylaminopropyl)-3-ethylcarbodiimide hydrochloride (EDC), N-Hydroxysulfosuccinimide sodium salt (NHS), Fluorescein isothiocyanate (FITC) and Indocyanine green (ICG) were supplied by Macklin. NH_2_-PEG-NH_2_ came from Carbohydrates Technology. Fetal bovine serum (FBS), Trypsin, RPMI-1640 medium, Streptomycin Penicillin, and Cell counting kit-8 (CCK-8) were purchased from Wilber. Lyso-Tracker Red Probe, Apoptosis Kit, NO Detection Kit, and ROS detection kit were provided by Beyotime. Cell Live Dead Staining Kit and ONOO^−^ Detection Kit were supplied by Bestbio. Hoechst 33342 came from Biosharp. ATP Detection Kit was obtained from Solarbio. 5,5′,6,6′-tetrachloro-1,1′,3,3′-tetraethylbenzimidazolylcarbocyanine iodide (JC-1), anti-CD11c-FITC, anti-CD80-APC, anti-CD86-PE, anti-CD3-FITC, anti-CD4-APC, anti-CD8a-PE, anti-CD86-PE, anti-CD206-PE, anti-F4/80-APC, anti-CD11b-FITC, and anti-CD11c-FITC were obtained from Elabscience. All ELISA kits (IFN-γ, IL-10, IL-12p40, GZMS-B, PGE_2_ and TNF-α) were purchased from Boster. 4T1 cells were provided by the cell bank of the Shanghai Institute of Biochemistry and Cell Biology, Chinese Academy of Sciences.

NMR spectra were measured using a superconducting nuclear magnetic resonance spectrometer AVANCE Ⅲ HD (Switzerland); Fluorescence emission spectra were recorded on a fluorescence spectrometer RF-5301pc (Japan); Absorption spectra were measured on a UV–visible spectrophotometer TU-1810 (China); High-resolution mass spectra were measured using a high-resolution time-of-flight mass spectrometer MicroTof Q II (USA); Fluorescence images of cells were taken using a fluorescence microscope Axio Imager.Z2 (Germany); Cell viability and cytokines were recorded using a microplate reader Flash3001 (USA); The morphology of the samples was analyzed by a scanning electron microscope JSM-5601LV (Japan); The structure of the samples was characterized using an X-ray diffractometer Smartlab-SE (Japan), an X-ray photoelectron spectrometer ESCALAB 250Xi (USA), and a Fourier transform infrared spectrometer VERTEX 70v (Germany); Zeta potential and particle size analyzer 90Plus Pals were used (USA) to characterize samples; Thermogravimetric changes of samples were recorded using a synchronous thermal analyzer STA449F3 (Germany); Temperature changes of samples and living bodies were recorded using an infrared thermal imager Fotric 325L (China); *In vivo* photoacoustic data were recorded using an ultrasonic imaging instrument Vevo F2 LAZR-X (Japan); *In vivo* fluorescence imaging data were recorded using an *in vivo* imager VISQUE Invivo Smart-LF (Singapore); Flow cytometry analysis was performed using a flow cytometer CytoFLEX (USA).

### 2.2 Synthesis process

#### 2.2.1 Synthesis of hollow copper sulfide nanoparticles (HCuSNPs)

CuCl_2_·2H_2_O solution (100 μL) and polyvinyl pyrrolidone (0.24 g) were mixed in deionized water (25 mL) and stirred at room temperature. NaOH (pH 9, 25 mL) solution was added to the stirred mixed solution, and then hydrazine solution (6.4 μL) was added. The color of the solution turned to bright yellow to form a Cu_2_O ball suspension. Subsequently, 200 μL of Na_2_S (320 mg mL^−1^) aqueous solution was added to the Cu_2_O suspension, stirred at 60 °C for 2 h to ensure sufficient reaction until the solution turned dark green, cooled to room temperature, centrifuged (11000 rpm, 10 min), washed 3 times with deionized water, and HCuSNPs were obtained by freeze-drying.

#### 2.2.2 Synthesis of NOSH

Compound 1: Under nitrogen, DCC (845 mg, 4.09 mmol), DMAP (50 mg, 0.409 mmol) and salicylaldehyde (500 mg, 4.09 mmol) were added to 4-bromobutyric acid (683 mg, 4.09 mmol) in CH_2_Cl_2_, and the whole reaction mixture was stirred at room temperature overnight. After the reaction was completed (checked by TLC), the precipitate was filtered off, and the organic phase was extracted into CH_2_Cl_2_ (2x15 mL) after adding water. The organic solvent was removed by reduced pressure to obtain the crude product. The product was purified by column chromatography with a yield of 80 %.

Compound 2: Compound 1 (500 mg, 1.84 mmol) was mixed with AgNO_3_ (780 mg, 4.6 mmol) in CH_3_CN (20 mL), stirred at 70 °C for 7 h, filtered through celite and concentrated under reduced pressure. The organic layer was separated after adding CH_2_Cl_2_ (20 mL) and H_2_O (20 mL), and the aqueous layer was extracted 3 times with CH_2_Cl_2_ (20 mL). All organic layers were dried and concentrated under reduced pressure. The crude product was purified by flash chromatography. Eluent [90:10 PE/EtOAc (v/v)]. The product was a light yellow oil with a yield of 84 %.

Compound 3: KMnO_4_ (692 mg, 4.38 mmol) was added to a stirred solution of Compound 2 (2.96 g, 11.74 mmol) in acetone (20 mL) at 0 °C. The reaction mixture was heated to room temperature and stirred for 3 h. After the reaction was completed (checked by TLC), oxalic acid was added and the precipitate was filtered off. The filtrate was diluted with dichloromethane, washed with water, dried, concentrated under reduced pressure, and the crude product was purified by crystallization to obtain the product. White solid, yield 83 %.

Compound NOSH: DCC (201.0 mg, 0.97 mmol) and DMAP (10.8 mg, 0.09 mmol) were added to CH_2_Cl_2_ of Compound 3 (238.0 mg, 0.88 mmol). Then ADT-OH (200.0 mg, 0.88 mmol) was added and stirred at room temperature overnight. After the reaction was completed by TLC, the precipitate was filtered off, water was added, and then extracted into dichloromethane (2x50 mL). The organic solvent was removed under reduced pressure to obtain a crude product. Further purification by column chromatography gave a pure orange solid with a yield of 78 %.

#### 2.2.3 Synthesis of NOSH@HCuSNPs

Take 10 mL of HCuSNPs (1 mg mL^−1^) in deionized water and stir with 1 mL of NOSH (10 mg mL^−1^) in DMSO at room temperature, and stir overnight. The mixture was centrifuged at 10000 g. The crude product was washed three times with DMSO. Finally, it was dispersed in deionized water for later use.

#### 2.2.4 Synthesis of NH_2_-PEG-FA

Folic acid FA (44.1 mg, 0.1 mmol) was dissolved in DMSO (3 mL). Then EDC·HCl (28.8 mg, 0.15 mmol) and hydroxysuccinimide NHS (17.3 mg. 0.15 mmol) were added to the above solution and stirred in the dark for 12 h. Finally, NH_2_-PEG-NH_2_ (400 mg) was dissolved in CHCI_3_ (1 mL) and DMSO (2 mL), and FA was added to the above reaction solution and stirred for 24 h. The product was precipitated in ether and dried under vacuum.

#### 2.2.5 Synthesis of NOSH@PEG-HCuSNPs

NH_2_-PEG-FA (200 mg) and NOSH@HCuSNPs (20 mg) were dissolved in 20 mL of deionized water and stirred at room temperature for 12 h. The material was washed three times with deionized water and freeze-dried. Finally, it was dispersed in deionized water for use.

#### 2.2.6 Synthesis of FITC-NOSH@PEG-HCuSNPs

FITC (0.1 mg mL^−1^) was mixed with an ethanol solution of NOSH@PEG-HCuSNPs (1 mg mL^−1^, 10 mL) and stirred at room temperature for 12 h. Subsequently, it was centrifuged and washed to obtain FITC-labeled NOSH@PEG-HCuSNPs, which were redispersed in deionized water for cell imaging.

#### 2.2.7 Synthesis of ICG-NOSH@PEG-HCuSNPs

ICG and NOSH@PEG-HCuSNPs were dissolved in 10 mL of ethanol solution at a ratio of 1:2 and vigorously stirred at room temperature for 12 h, followed by centrifugation. After washing with ethanol, ICG-labeled NOSH@PEG-HCuSNPs were obtained and finally redispersed in deionized water for *in vivo* imaging.

### 2.3 In vitro performance characterization

#### 2.3.1 *In vitro* photothermal performance

First, the NOSH@PEG-HCuSNP deionized water solutions with different concentrations (0, 25, 50, 100, 200, 400 and 800 mg L^−1^) in the EP tube were irradiated with an 808 nm near-infrared laser with a power density of 1.5 W cm^−2^ for 10 min. Secondly, the NOSH@PEG-HCuSNP deionized water solution with a concentration of 200 mg L^−1^ was irradiated with a near-infrared laser with several power densities (0, 0.5, 1.0, 1.5 and 2.0 W cm^−2^) for 10 min. Finally, in order to investigate the thermal stability of NOSH@PEG-HCuSNP, the solution was irradiated with an 808 nm laser (1.5 W cm^−2^) for 10 min, and then cooled naturally for 15 min. The temperature change curves of five consecutive cycles were recorded, and the photothermal conversion efficiency (η) was evaluated according to the previously reported formula. Specifically,The photothermal conversion efficiency was calculated using the following equation:

Among them, hAs (Tmax−Ts): Total heat generated from photothermal conversion; Q_dis_: Heat dissipation power from non-photothermal processes, estimated from the control experiments.

#### 2.3.2 *In vitro* NO release assay

We chose the GRIESS reagent method as the method to detect the release of NO in NOSH@PEG-HCuSNPs. The GRIESS reagent was prepared as follows: 6 mL of concentrated phosphoric acid (85 %), 70 mL of deionized water and 1.0 g of anhydrous p-aminobenzenesulfonic acid were fully dissolved and then diluted to 100 mL to prepare reagent A; 0.1 g of N-1-naphthylethylenediamine hydrochloride was dissolved in hydrogen ion water and diluted to 100 mL to prepare reagent B. During the detection process, reagent A and reagent B were mixed in a ratio of 1:1 to prepare the GRIESS reagent for detection.

First, the standard curve of GRIESS reagent for detecting NO was drawn by adding different concentrations of NaNO_2_ (0, 12.5, 25, 50, 100, 200, 400, 800 μM). Then, NOSH@PEG-HCuSNPs and GRIESS reagent were added to PBS solution at the same time. By adding different concentrations (0, 25, 50, 100, 200, and 400 μg mL^−1^) of NOSH@PEG-HCuSNPs and laser irradiation of different powers (0, 0.5, 0.75, 1, 1.25, and 1.5 W cm^−2^), the UV absorption at 540 nm was recorded by UV spectrophotometer after a period of time under different pretreatment conditions. Finally, the amount of NO released under different pretreatment conditions was calculated by the standard curve.

#### 2.3.3 *In vitro* H_2_S release assay

We selected probe NP-N_3_ as the detection of H_2_S release in NOSH@PEG-HCuSNPs. First, by adding different concentrations (0, 0.25, 0.5, 1, 2, 5, 10, 20, 40 μM) of NaHS, the standard curve of probe NP-N_3_ for detecting H_2_S was drawn. Then, NOSH@PEG-HCuSNPs and NP-N_3_ were added to DMSO: PBS (v/v = 4:1) solution at the same time. By adding different concentrations (0, 25, 50, 100, 200, and 400 μg mL^−1^) of NOSH@PEG-HCuSNPs and laser irradiation of different powers (0, 0.5, 0.75, 1, 1.25, and 1.5 W cm^−2^), the fluorescence emission intensity at the emission wavelength of 520 nm under different pretreatment conditions was recorded by fluorescence spectrophotometer. Finally, the amount of H_2_S released under different pretreatment conditions was calculated by the standard curve.

#### 2.3.4 Stability evaluation

NOSH@PEG-HCuSNPs were dispersed in Aqueous, PBS, Normal Saline and 10 % FBS 1640 medium to evaluate their biostability under different simulated environments. The experimental solution was kept at 37 °C and stirred at 100 rpm to simulate the *in vivo* environment. Then, the experimental solution was collected from each sample at a given time, and the biodegradation behavior of NOSH@PEG-HCuSNPs was directly studied by transmission electron microscopy and the particle size change of the nanomaterials was recorded.

NOSH@PEG-HCuSNPs were incubated in PBS solution and 10 % FBS 1640 medium under 808 nm laser irradiation, and TEM images after different incubation times were observed by transmission electron microscopy.

### 2.4 Cell experiment

#### 2.4.1 Cell culture

Breast cancer cells 4T1 were used for cell verification and *in vivo* modeling experiments in this study. 4T1 cells were cultured at 37 °C in a humidified atmosphere containing 5 % carbon dioxide (CO_2_) using RPMI-1640 medium supplemented with 10 % fetal bovine serum (FBS) and 1 % streptomycin/penicillin.

#### 2.4.2 Cytotoxicity

The cytotoxicity of the synthesized materials was evaluated using the CCK-8 method. First, 4T1 cells were inoculated in a 96-well plate with 5000 cells per well and cultured in RPMI-1640 medium containing 10 % FBS at 37 °C and 5 % CO_2_ for 12 h. Then, different concentrations of HCuSNP, NOSH and NOSH@HCuSNPs were added and incubated for 12 h, 24 h and 48 h, respectively. Subsequently, CCK-8 (10 μL) solution was added to each well and further incubated for 2 h. The absorbance value of each well was measured at 450 nm using a microplate reader, and there were 6 parallel groups for each concentration. The cell survival rate was calculated by VR = A/A_0_ × 100 %, where A was the absorbance value of the experimental group at different concentrations, and A_0_ was the UV absorbance value of the blank control group.

#### 2.4.3 Cell uptake

4T1 cells were seeded in 12-well plates and incubated with RPMI-1640 medium containing 10 % FBS for 24 h under standard conditions (37 °C, 5 % CO_2_). Then, Hoechst 33342 dye for labeling cell nuclei, Lyso-Tracker Red dye for labeling lysosomes, and FITC-NOSH@PEG-HCuSNPs for labeling materials were added to the cells. The cells were incubated for appropriate times (0, 0.5 h, 1 h, 2 h, and 4 h). Fluorescence microscopy and flow cytometry were used to observe the uptake of materials by cells.

#### 2.4.4 Anticancer activity

4T1 cells were seeded in 96-well plates, with 5000 cells per well, and cultured in RPMI-1640 medium containing 10 % FBS at 37 °C and 5 % CO_2_ for 12 h. The cells were grouped and pretreated with different treatments: (1) Blank, (2) Laser (1.0 W cm^−2^), (3) HCuSNPs, (4) NOSH, (5) NOSH@PEG-HCuSNPs, (6) HCuSNPs + Laser(1.0 W cm^−2^), (7) HCuSNPs + Laser(0.5 W cm^−2^), (8) NOSH@PEG-HCuSNPs + Laser(1.0 W cm^−2^) and (9) NOSH@PEG-HCuSNPs + Laser(0.5 W cm^−2^). The experimental group without laser treatment was directly incubated for 12 h after administration, and the group requiring laser treatment was irradiated with 808 nm laser for 10 min and then continued to be incubated for 12 h. All experimental groups had 6 parallel groups, and the anti-tumor activity was evaluated by CCK-8 method.

#### 2.4.5 Live and dead cell staining

The Calcein-AM/PI live and dead cell staining kit was used to detect cell apoptosis. 4T1 cells were seeded in 12-well plates and incubated with RPMI-1640 medium containing 10 % FBS for 24 h under standard conditions (37 °C, 5 % CO_2_). The cells were grouped and pretreated differently: (1) Blank, (2) Laser (1.0 W cm^−2^), (3) HCuSNPs, (4) NOSH, (5) NOSH@PEG-HCuSNPs, (6) HCuSNPs + Laser(1.0 W cm^−2^), (7) HCuSNPs + Laser(0.5 W cm^−2^), (8) NOSH@PEG-HCuSNPs + Laser(1.0 W cm^−2^) and (9) NOSH@PEG-HCuSNPs + Laser(0.5 W cm^−2^). The laser-treated group was irradiated with 808 nm laser for 10 min. Afterwards, the cells were moved to standard conditions (37 °C, 5 % CO_2_) for 1 h, washed twice with pre-cooled PBS, and incubated with Calcein-AM and PI double staining reagent for 15 min. After washing the cells with PBS, the cells were immediately imaged by fluorescence microscopy.

#### 2.4.6 Apoptosis

Annexin V-FITC/PI apoptosis detection kit was used to detect cell apoptosis. 4T1 cells were seeded in 12-well plates and incubated with RPMI-1640 medium containing 10 % FBS for 24 h under standard conditions (37 °C, 5 % CO_2_). The cells were grouped and subjected to different pretreatments: (1) Blank, (2) Laser (1.0 W cm^−2^), (3) HCuSNPs, (4) NOSH, (5) NOSH@PEG-HCuSNPs, (6) HCuSNPs + Laser(1.0 W cm^−2^), (7) HCuSNPs + Laser(0.5 W cm^−2^), (8) NOSH@PEG-HCuSNPs + Laser(1.0 W cm^−2^) and (9) NOSH@PEG-HCuSNPs + Laser(0.5 W cm^−2^). The group requiring laser treatment was irradiated with 808 nm laser for 10 min. Afterwards, the cells were moved to standard conditions (37 °C, 5 % CO_2_) for 1 h, washed twice with pre-cooled PBS, and then resuspended in 100 μL of PBS buffer. The proportion of apoptotic cells was detected by flow cytometry using the method described in the Annexin V-FITC/PI kit.

#### 2.4.7 Detection of mitochondrial membrane potential

The JC-1 mitochondrial membrane potential detection kit was used to observe the changes in mitochondrial membrane potential in 4T1 cells after different pretreatments. 4T1 cells were seeded in 12-well plates and incubated with RPMI-1640 medium containing 10 % FBS for 24 h under standard conditions (37 °C, 5 % CO_2_). The cells were grouped and subjected to different pretreatments: (1) Blank, (2) Laser (1.0 W cm^−2^), (3) HCuSNPs, (4) NOSH, (5) NOSH@PEG-HCuSNPs, (6) HCuSNPs + Laser(1.0 W cm^−2^), (7) HCuSNPs + Laser(0.5 W cm^−2^), (8) NOSH@PEG-HCuSNPs + Laser(1.0 W cm^−2^) and (9) NOSH@PEG-HCuSNPs + Laser(0.5 W cm^−2^). The laser-treated group was irradiated with 808 nm laser for 10 min. Afterwards, the cells were moved to standard conditions (37 °C, 5 % CO_2_) for 1 h and then incubated with JC-1 working solution at 37 °C for 30 min. Subsequently, the cells were washed with PBS, and the changes in mitochondrial membrane potential in the cells were observed by fluorescence microscopy (red fluorescence, ex = 585 nm, em = 590 nm; green fluorescence, ex = 514 nm, em = 529 nm).

#### 2.4.8 Intracellular H_2_S, NO, ROS and ONOO^−^ detection

NP-N_3_ probe, DAF-FM DA kit, DCFH-DA probe and ONOO^−^ detection kit were used to detect changes in intracellular H_2_S, NO, ROS and ONOO^−^ content. 4T1 cells were seeded in 12-well plates and incubated with RPMI-1640 medium containing 10 % FBS for 24 h under standard conditions (37 °C, 5 % CO_2_). The cells were grouped and pretreated differently: (1) Blank, (2) Laser (1.0 W cm^−2^), (3) HCuSNPs, (4) NOSH, (5) NOSH@PEG-HCuSNPs, (6) HCuSNPs + Laser(1.0 W cm^−2^), (7) HCuSNPs + Laser(0.5 W cm^−2^), (8) NOSH@PEG-HCuSNPs + Laser(1.0 W cm^−2^) and (9) NOSH@PEG-HCuSNPs + Laser(0.5 W cm^−2^). The laser-treated group was irradiated with 808 nm laser for 10 min. Afterwards, the cells were incubated with different working solutions at 37 °C according to the instructions of different probes and kits. After washing the cells with PBS, the cell images were immediately recorded by fluorescence microscopy.

#### 2.4.9 ATP detection

The ATP content in 4T1 tumor cells was determined using an ATP detection kit. 4T1 cells were seeded in 6-well plates and incubated with RPMI-1640 medium containing 10 % FBS for 24 h under standard conditions (37 °C, 5 % CO_2_). The cells were divided into groups and subjected to different pretreatments: (1) Blank, (2) Laser (1.0 W cm^−2^), (3) HCuSNPs, (4) NOSH, (5) NOSH@PEG-HCuSNPs, (6) HCuSNPs + Laser(1.0 W cm^−2^), (7) HCuSNPs + Laser(0.5 W cm^−2^), (8) NOSH@PEG-HCuSNPs + Laser(1.0 W cm^−2^) and (9) NOSH@PEG-HCuSNPs + Laser(0.5 W cm^−2^). The laser-treated groups were irradiated with 808 nm laser for 10 min. Afterwards, ATP content was determined according to the ATP Assay Kit procedure. Operate on ice, add 500 μL of extract to each well, ultrasonically disrupt, and centrifuge. Take the supernatant and add 500 μL of chloroform to mix, centrifuge, and collect the supernatant. Use an enzyme reader to measure the absorbance value at 340 nm, and calculate the ATP content in each group through the standard curve.

#### 2.4.10 Western blotting

4T1 cells were inoculated in a 6-well plate and incubated with RPMI-1640 medium containing 10 % FBS for 24 h under standard conditions (37 °C, 5 % CO_2_). The group to be laser treated was irradiated with 808 nm laser for 10 min. Wash the cells three times with cold PBS, and lyse the cells after 12 h to determine the total protein using the BCA protein assay kit. The same mass of protein (40 μg) was separated by 12 % or 10 % tris-succinyl gel electrophoresis, and then the separated proteins were transferred to polyvinylidene difluoride membrane (PVDF) and sealed with 5 % skim milk for 1 h to reduce nonspecific binding. Finally, the primary and secondary antibodies were used for detection, and the enhanced chemiluminescence detection kit was used for analysis in a chemiluminescence imaging system. Western blotting was used to detect the expression levels of heat shock protein 90 (Hsp 90) and cytochrome *c* oxidase subunit IV (COX IV) in cells after different treatments.

#### 2.4.11 mRNAs profiling analysis

Total RNA was extracted using TRIzol reagent according to the instructions. RNA purity and quantification were identified using NanoDrop 2000 spectrophotometer, and RNA integrity was assessed using Agilent 2100 Bioanalyzer. Transcriptome libraries were constructed using VAHTS Universal V5 RNA-seq Library Prep Kit according to the instructions. Transcriptome sequencing and analysis were performed by Shanghai Ouyi Biotechnology Co., Ltd.

First, the Illumina Novaseq 6000 sequencer was used to obtain raw reads data after image recognition and base recognition. Next, fastp software was used to remove adapters and low-quality reads to obtain high-quality clean reads for subsequent data analysis. We used DESeq2 software to perform differentially expressed gene analysis, where genes that met the thresholds of q value < 0.05 and foldchange >2 or foldchange <0.5 were defined as differentially expressed genes (DEGs). GO and KEGG pathway analysis were performed on host genes of differentially expressed mRNA to show the expression changes of up-regulated or down-regulated genes. The Search Tool for The Retrieval of Interacting Genes/Proteins (STRING) algorithm was used to analyze the functional interaction network of regulatory proteins involved in signaling pathways.

### 2.5 In vivo experiment

#### 2.5.1 Hemolysis test

Blood was collected from mice by orbital venous plexus blood sampling and sodium heparin was added. Red blood cells were separated from the blood samples by centrifugation. 1 mL of separated red blood cells was diluted in 50 mL PBS and treated with the same volume of NOSH@PEG-HCuSNPs at 37 °C for 60 min. In addition, DI water and PBS were added to the same volume of diluted separated red blood cells as positive and negative controls, respectively. After incubation, all samples were centrifuged at 1000 rpm for 3 min, 100 μL of supernatant was transferred to a 96-well plate, and the absorbance at a wavelength of 590 nm was measured using an ELISA reader.

#### 2.5.2 *In vivo* biosafety evaluation

Healthy male Balb/c mice (six weeks old, n = 4) were randomly divided into groups for the experiment. Each mouse was injected with a single dose of PBS, HCuSNPs, and NOSH@PEG-HCuSNPs through the tail vein at a dose of 20 mg kg^−1^. Mice were killed on the 14th day after administration. Then, the mice were dissected, and the main tissues and organs (heart, liver, spleen, lung, and kidney) were removed, fixed with 4 % paraformaldehyde, embedded in paraffin, and tissue sections were prepared. These tissue sections will be stained with hematoxylin and eosin (H&E) to observe pathological characteristics, evaluate the possible toxicity of combined treatment on major organs, and capture images.

#### 2.5.3 Establishment of mouse tumor model

Male Balb/c mice were purchased from Lanzhou Veterinary Research Institute, Chinese Academy of Agricultural Sciences. All experiments were carried out in accordance with the guidelines of the Ethics Committee of Lanzhou Institute of Chemical Physics, Chinese Academy of Sciences, and the experiments were approved by the Ethics Committee of Lanzhou Institute of Chemical Physics, Chinese Academy of Sciences.

Primary tumor model: 4T1 tumor cells (2 × 10^6^ cells) suspended in PBS (100 μL) were subcutaneously injected into the right axilla of mice to establish a tumor model. The tumor was allowed to grow for about 18 days, and the experiment was performed when the tumor volume reached about 100 mm^3^.

Double tumor model: 4T1 tumor cells (2 × 10^6^ cells) suspended in PBS (100 μL) were subcutaneously injected into the right axilla of mice. When the tumor volume reached about 50 mm^3^, 4T1 tumor cells (2 × 10^6^ cells) suspended in PBS (50 μL) were subcutaneously injected into the left axilla of mice. The experiment was started when the right axillary tumor volume of all mice reached about 100 mm^3^.

#### 2.5.4 *In* vivo imaging

Fluorescence imaging: ICG-NOSH@PEG-HCuSNPs (200 μL, 20 mg kg^−1^) modified with ICG were injected into tumor-bearing mice (n = 4) through the tail vein, and then the fluorescence images at 0, 1, 3, 6, 12, and 24 h were captured using an *in vivo* fluorescence imager.

Photoacoustic imaging: NOSH@PEG-HCuSNPs (200 μL, 20 mg kg^−1^) were injected into tumor-bearing mice (n = 4) through the tail vein, and the photoacoustic signals of the respective tumor areas of the mice administered at different time points (0, 1, 3, 6, 12, 24 h) were recorded.

#### 2.5.5 *In vivo* photothermal performance

PBS (200 μL), HCuSNPs (200 μL, 20 mg kg^−1^) and NOSH@PEG-HCuSNPs (200 μL, 20 mg kg^−1^) were injected into tumor-bearing mice (n = 4) through the tail vein. 6 h after injection, the mouse tumor was irradiated with 808 nm laser (0.5 W cm^−2^/1.0 W cm^−2^) for 10 min. The tumor temperature was monitored and the infrared thermal image was recorded using an infrared thermal imager.

#### 2.5.6 *In vivo* antitumor effect

The tumor-bearing mice were randomly divided into 9 groups, namely (1) Blank, (2) Laser (1.0 W cm^−2^), (3) HCuSNPs, (4) NOSH, (5) NOSH@PEG-HCuSNPs, (6) HCuSNPs + Laser(1.0 W cm^−2^), (7) HCuSNPs + Laser(0.5 W cm^−2^), (8) NOSH@PEG-HCuSNPs + Laser(1.0 W cm^−2^) and (9) NOSH@PEG-HCuSNPs + Laser(0.5 W cm^−2^). Laser (808 nm, 10 min) was applied 6 h after administration. The tumor volume and mouse weight of each group were measured every 3 days to evaluate the treatment results. Tumor volume (V mm^3^) = (a × b^2^)/2, where a and b are the length and width of the tumor, respectively.

#### 2.5.7 Immune response evaluation

The mice in each group were dissected after treatment, and the tumors, spleens, and tumor-draining lymph node tissues of the mice were collected and dissociated into single cell suspensions using tissue lysis buffer. After centrifugation, the cells were resuspended in culture medium and washed with PBS, stained at room temperature for 1 h, and finally the cell content was detected by flow cytometry. In the experiment, different antibodies were used to stain different cell types, among which DC cells were stained with anti-CD11c-FITC, anti-CD80-APC, and anti-CD86-PE antibodies; for T cells, anti-CD3-FITC, anti-CD4APC, and anti-CD8a-PE antibodies were used for staining; M1 macrophages/M2 macrophages were stained with anti-CD86-PE/anti-CD206-PE, anti-F4/80-APC, and anti-CD11bFITC antibodies. In addition, tumor sections of each group were further studied by hematoxylin and eosin (H&E) staining, TUNEL (TdT-mediated dUTP Nick-End Labeling) method and ki67 immunohistochemical staining. Serum samples of mice after various treatments were collected and diluted for analysis. Cytokines INF-γ, TNF-α, GZMS-B, IL-12p40, IL-10, and PGE_2_ were analyzed using ELISA kits according to the supplier's protocol.

### 2.6 Statistical analysis

The data obtained in the experiment were verified at least 3 times and are expressed as mean ± standard deviation (SD). One-way ANOVA was performed using SPSS statistical software to determine multiple comparisons between groups. ∗∗∗P < 0.001, ∗∗P < 0.01, and ∗P < 0.05 were considered statistically significant.

## 3 Results

### 3.1 Design, synthesis, and characterization of NOSH and NOSH@PEG-HCuSNPs

**Scheme 1:**
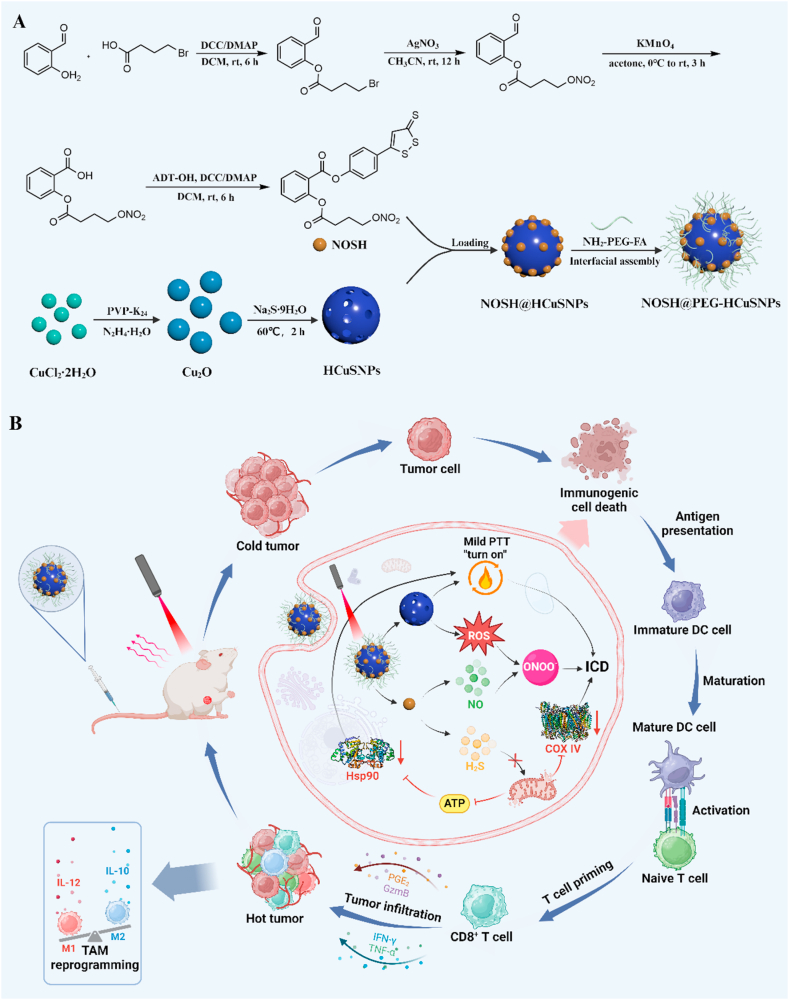
(A) Construction process of NOSH@PEG-HCuSNPs. (B) Schematic diagram of NIR-triggered programmable NOSH@PEG-HCuSNPs for cascading oncotherapy by mPTT/gas/ROS-reinforced ICD.

In order to synthetize the gas donor NOSH with NO and H_2_S, as shown in Scheme 1, the nitrate (-ONO_2_) groups as donors were employed to release NO and then attach them to salicylaldehyde by esterification. Afterwards, the H_2_S releasing group 5-(4-hydroxyphenyl)-3H-1,2-dithiole-3-thione (ADT-OH) was also directly coupled to the salicylaldehyde skeleton via esterification to obtain NOSH [34]. The structure of NOSH was characterized by ^1^H NMR, ^13^N NMR, and high-resolution mass spectrometry. The spectra shown in Figure S1 and Figure S2 showed the characteristic peaks of NOSH, and Fig. S3 showed the characteristic peak of M + Na at *m*/*z* 499.99, which means that the synthesis was successful.

**Fig. 1:**
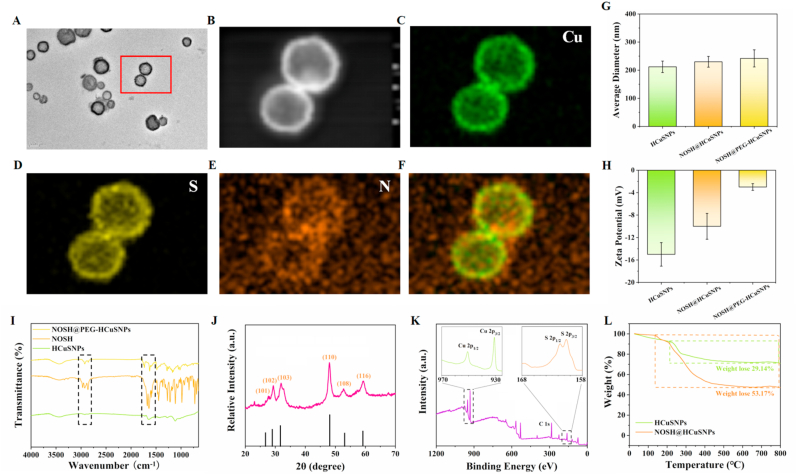
**Physicochemical characterization of HCuSNPs, NOSH@HCuSNPs, and NOSH@PEG-HCuSNPs.** (A) TEM images and (B–F) TEM-mapping images of NOSH@PEG-HCuSNPs. (G–I) Size distribution, ζ potential, and FT-IR spectra of HCuSNPs, NOSH@HCuSNPs, and NOSH@PEG-HCuSNPs. (J-K)XRD spectra and High-resolution XPS spectra of NOSH@PEG-HCuSNPs. (L) TGA curves of HCuSNPs, NOSH@HCuSNPs, and NOSH@PEG-HCuSNPs. Data are expressed as the mean ± SD.

Next, hollow mesoporous copper sulfide nanoparticles (termed as HCuSNPs) were synthesized by a simple two-step synthesis procedure according to previous literature reports [32]. Afterwards, enlightened by the interfacial assembly, the gas donor NOSH was loaded into the hollow nanostructure of HCuSNPs and then modified by a polyethylene glycol derivative containing with folic acid (NH_2_-PEG_2000_-FA) to obtain NIR-triggered programmable targeting nanomotor (termed as NOSH@PEG-HCuSNPs) (Scheme 1). As shown in Fig. 1A and Fig. S4**, the field emission transmission electron microscopy (TEM) showed that as-constructed NOSH@PEG-HCuSNPs had an appropriate spherical with hollow mesoporous cavities. In addition, mapping analysis illustrated the uniform distribution of Cu, S, and N elements in the hollow NOSH@PEG-HCuSNPs framework (**Fig. 1B–F). As observed from the dynamic light scattering (DLS), the mean diameters of HCuSNPs, NOSH@HCuSNPs, and NOSH@PEG-HCuSNPs were 212.1, 230.2, and 242.0 nm, respectively, which was well consistent with TEM results (Fig. 1G). The ζ potentials of HCuSNPs, NOSH@HCuSNPs, and NOSH@PEG-HCuSNPs were −15.1 ± 2.1, −10.3 ± 2.3, and −3.3 ± 0.6 mV, respectively (Fig. 1H). It should be pointed out that these changes in diameter and ζ potential might be mainly resulted in HCuSNPs loaded and modified via positively charged NOSH and NH_2_-PEG_2000_-FA.

In order to further confirm whether gas donor NOSH was successfully loaded into HCuSNPs, Fourier transform infrared (FT-IR) spectroscopy analysis, the powder X-ray diffraction (XRD) pattern, and X-ray photoelectron spectroscopy (XPS) were conducted. As depcited in the FT-IR of Fig. 1I, the constructed NOSH@PEG-HCuSNPs showed unique characteristic peaks of NOSH, such as the characteristic peak of R_2_CH_2_ group at around 1700 cm^−1^ and the characteristic peak of ester bond at around 2900 cm^−1^. As shown in Fig. 1J, the characteristic peaks at 2 *θ* = 26.76, 29.58, 31.86, 48.07, 52.85, and 59.18 corresponds to the crystal planes (101), (102), (103), (108), (110) and (116) of the hexagonal Cu crystal, respectively. Notably, the XRD spectra of HCuSNPs, NOSH@HCuSNPs, and NOSH@PEG-HCuSNPs possessed the similar Cu crystal structures (Fig. S5) In order to further clarify the formation mechanism of NOSH@PEG-HCuSNPs, the chemical states and binding energies of the associated elements were detected. As shown in Fig. 1K, the chemical states of Cu and S were analyzed by XPS. The peaks at 930.8 and 950.7 eV were attributed to the binding energies of Cu 2p3/2 and Cu 2p1/2, and the peaks observed in the range of 158–168 eV corresponded to the binding energies of S2p3/2 and S2p1/2. These above results were consistent with the XPS data of CuS nanocrystals reported previously [35]. Moreover, the effective payload of NOSH was confirmed by thermogravimetric analysis (TGA) of HCuSNPs and NOSH@HCuSNPs (Fig. 1L). The payload of the gas donor NOSH was determined to be 24.03 %. In a word, the above results are consistent with the successful construction of NOSH@PEG-HCuSNPs.

Besides, in order to evaluate the physiological stability of NOSH@PEG-HCuSNPs, they were dispersed in water phosphate-buffered saline (PBS), saline, and 10 % FBS for one week to monitor size and morphology fluctuations. As could be seen in Fig. S6, NOSH@PEG-HCuSNPs showed no obvious size and morphology changes, evidencing that they had a good physiological stability. It was also found that 200 μg mL^−1^ of NOSH@PEG-HCuSNPs had a low hemolysis, indicating that they had an good biocompatibility and can be used in further biological applications (Fig. S7).

### 3.2 The photothermal effect induced by NOSH@PEG-HCuSNPs

**Fig. 2:**
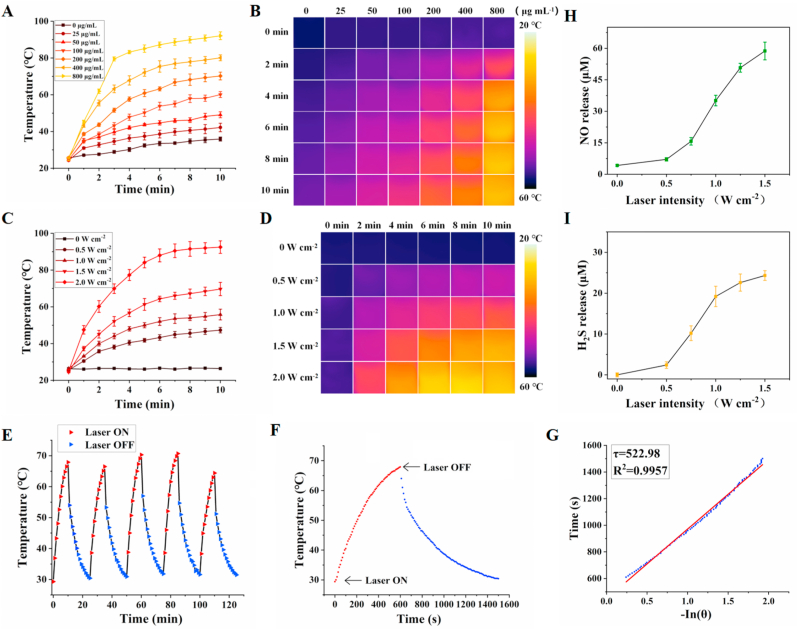
***In vitro* property valuation of NOSH@PEG-HCuSNPs**. (A) Photothermal effect and (B) Infrared thermal images of NOSH@PEG-HCuSNPs with different concentrations (0–800 μg mL^−1^) under 808 nm laser irradiation (1.5 W cm^−2^). (C) Photothermal effect and (D) infrared thermal images of NOSH@PEG-HCuSNPs (200 μg mL^−1^) under 808 nm laser irradiation with different laser intensities (0–2.0 W cm^−2^). (E) Temperature change curve of NOSH@PEG-HCuSNPs (200 μg mL^−1^) under repeated 808 nm laser irradiation for 5 cycles. (F) Temperature curve of NOSH@PEG-HCuSNPs (laser turned off after 10 min). (G) Time constant calculated from the cooling period. (H–I) NO and H_2_S release amount of NOSH@PEG-HCuSNPs (100 μg mL^−1^) under different 808 nm laser intensities (0–1.5 W cm^−2^) for 10 min. All experiments were performed at least three times. Data are expressed the mean ± SD (*n* = 3).

As shown in Fig. S8, the as-prepared NOSH@PEG-HCuSNPs have strong near-infrared absorption ability, indicating that NOSH@PEG-HCuSNPs have high application potential in NIR laser photothermal conversion. *In vitro* photothermal performance of NOSH@PEG-HCuSNPs under 808 nm laser irradiation dispersed in water was measured by an infrared thermal imager. The aqueous dispersions of NOSH@PEG-HCuSNPs with different concentrations were exposed to laser (1.5 W cm^−2^) for 10 min, and the results showed a concentration-dependent change trend. Unlike the blank group, which was insensitive to the thermal response from 808 laser irradiation, the temperature of NOSH@PEG-HCuSNPs in the aqueous solution at 200 μg mL^−1^ increased tp *ca.* 45 °C within 10 min (Fig. 2A). As depicted in Fig. 2B, a similar temperature increase trend could also be observed in the centrifuge tube through real-time thermal imaging image recording. In addition, the temperature change of NOSH@PEG-HCuSNPs in aqueous solution under different laser intensity was quantified (Fig. 2C). At a specific concentration (200 μg mL^−1^), the temperature of NOSH@PEG-HCuSNPs in aqueous solution could be adjusted from 47.3 °C to 92.5 °C when the irradiation power varied from 0.5 W cm^−2^ to 2.0 W cm^−2^. The change of the thermal imaging image in Fig. 2D also further evidence that the photothermal conversion performance of NOSH@PEG-HCuSNPs was highly dependent on the laser intensity density. In order to explore the photothermal stability of NOSH@PEG-HCuSNPs, the temperature change was monitored during the consecutive cycles of 5 laser on/off. As shown in Fig. 2E, the photothermal performance of NOSH@PEG-HCuSNPs didn't show obvious degradation under the repeated 808 nm laser irradiation, indicating that NOSH@PEG-HCuSNPs had an excellent photothermal stability. According to the calculation formula of the previous report [36], the photothermal conversion efficiency (*η*) of NOSH@PEG-HCuSNPs nanoparticles was also calculated to be *η* = 27.8 % based on the measured data in Fig. 2F and G. This value is slightly lower than some nanomaterials with photothermal conversion function in the NIR-I window, but it can meet the conditions for photothermal therapy [37,38]. Therefore, the above results indicated the feasibility of NOSH@PEG-HCuSNPs as an ideal photothermal agent with potential thermal therapy.

### 3.3 *In vitro* NO and H_2_S release of NOSH@PEG-HCuSNPs

According to the design strategy of NOSH@PEG-HCuSNPs, the loaded gas donor can simultaneously release H_2_S and NO. In order to ensure the effect on laser-mediated NO and H_2_S-assisted PTT, the NO and H_2_S release of gas donor NOSH in NOSH@PEG-HCuSNPs should be measured. The morphological changes of NOSH@PEG-HCuSNPs under 808 nm laser irradiation was observed and recorded by TEM. As shown in Fig. S9, after 808 nm laser irradiation of PBS or 10 % FBS culture medium for 12 h, it was found that the framework of NOSH@PEG-HCuSNPs was collapsed and its morphology was almost disappeared, meaning that laser-mediated NO and H_2_S release could be accelerated in NOSH@PEG-HCuSNPs.In order to further verify the NO release of NOSH@PEG-HCuSNPs, the Griess reagent was chosen as the detection method. As shown in Fig. S10, the nitrate (-ONO_2_) group could continuously release NO when dissolved in water. Therefore, in order to quantify the NO release from NOSH@PEG-HCuSNPs, the Griess reagent standard curve was established [31]. As shown in Fig. S11, under 808 nm laser irradiation, the release of NO continued to increase with the increase of laser treatment time, temperature and NOSH@PEG-HCuSNPs concentration. In addition, the NO release amount of NOSH@PEG-HCuSNPs treated with different laser intensities also had an obvious difference (Fig. 2H).

Next, the release of the H_2_S in NOSH@PEG-HCuSNPs was further evaluated by the H_2_S-specific fluorescent probe NP-N_3_ (this probe was synthesized according to previous work [39], the detection mechanism is shown in Fig. S12). Unlike other H_2_S donors, the exact mechanism of H_2_S release from ADT-OH and its derivatives is still unclear. However, it is hypothesized that cellular enzymes can lead to the H_2_S release from ADT-OH. In addition, previous studies have confirmed that ADT-OH can release H_2_S in rat liver homogenate, rat plasma, and cell lysate, but rarely releases H_2_S in buffer alone [5]. Based on this assumption, TCEP is usually added to promote the release of H_2_S from ADT-OH and its derivatives to achieve *in vitro* release detection. Based on the standard curve of NP-N_3_ for detecting H_2_S (Fig. S13), it can be found in Figs. S14 and 2I that the release amount of H_2_S in NOSH@PEG-HCuSNPs significantly changed and the photothermal effect further enhanced the release of H_2_S under different NOSH@PEG-HCuSNPs concentrations and different laser intensity irradiation. The increase in NOSH@PEG-HCuSNPs concentration and laser intensity led to an increase in the temperature in the aqueous solution, which promoted the hydrolysis process of the donor NOSH, enabling it to release NO and H_2_S faster and more.

In addition, the *in vitro* release experiment conducted above also proves that the donor NOSH can avoid the problem of premature release. The release of NO and H_2_S under non-laser triggered conditions showed that the release rate of NOSH from NOSH@PEG-HCuSNPs was still small without 808 nm laser irradiation, with less than 10 % released within 12 h, indicating strong retention under physiological conditions. This can be attributed to the stabilizing effect of PEG modification, which minimizes premature release during systemic circulation.

These findings collectively indicate that NOSH@PEG-HCuSNPs are able to maintain stability and minimize premature release under physiological conditions while ensuring effective release of therapeutic gas molecules at the tumor site after laser activation. This shows the operational feasibility of the proposed GT/PTT combination therapy.

### 3.4 *In vitro* anti-tumor and molecular mechanism of NOSH@PEG-HCuSNPs

**Fig. 3:**
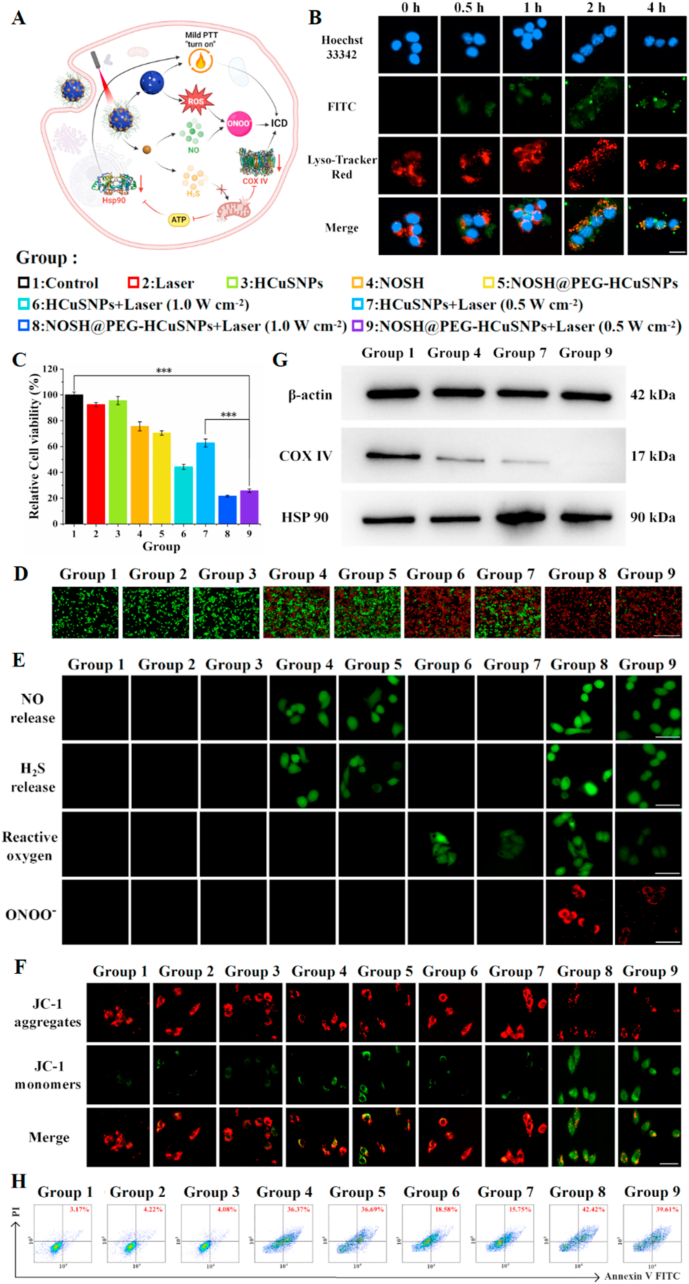
***In vitro* cellular experiments of NOSH@PEG-HCuSNPs.** (A) Schematic illustration of NOSH@PEG-HCuSNPs for cascading oncotherapy by GT and mPTT. (B) CLSM images of cellular endocytosis of FITC-labeled NOSH@PEG-HCuSNPs in 4T1 cells at different time points (green channel: FITC, red channel: Lyso-Tracker Red, blue channel: Hoechst 33342, scale: 20 μm). (C) cell viability and (D) Calcein-AM/PI staining images (green channel: Calcein AM, red channel: PI, scale: 500 μm) of 4T1 cells after different treatments. (E) The staining images of DAF-FM DA kit, NP-N_3_ probe, DCFH-DA probe, and ONOO^−^ detection kit was employed to evaluate the NO release, H_2_S release, ROS release, and ONOO^−^ generation after different treatments (scale: 20 μm), respectively. (F) The JC-1 staining images (green channel: JC-1 monomer, red channel: JC-1 aggregate, scale: 20 μm) of 4T1 cells after different treatments. (G) COX IV and HSP 90 expressions and (H) Flow cytometry after Annexin V-FITC/PI staining of 4T1 cells after different treatments. Data are expressed as the mean ± SD (*n* = 3) and ∗∗∗P < 0.001. (For interpretation of the references to color in this figure legend, the reader is referred to the Web version of this article.)

Encouraged by the good physicochemical properties, the anti-tumor effect and the associated molecular mechanism of NOSH@PEG-HCuSNPs were further evaluated and investigated on a cellular level (Fig. 3A). In detail, NOSH@PEG-HCuSNPs were labeled with Fluorescein Isothiocyanate (FITC) probe, and their intracellular uptake efficiency was qualitatively evaluated by confocal laser scanning microscopy (CLSM). As shown in Fig. 3B, the fluorescence intensity of FITC in 4T1 cells obviously increased upon time prolonging from 0 h to 4 h, and reached a peak value at 1 h, indicating that NOSH@PEG-HCuSNPs could be quickly and effectively internalized into 4T1 cells. Notably, the co-localization tendency of lysosomes and NOSH@PEG-HCuSNPs gradually increased. It should be pointed out that the above mentioned qualitative CLSM results were well line with the quantitative flow cytometry (Fig. S15). These phenomena could be explained by the mature theory that folic acid (FA)-modified nanoparticles could be specifically internalized into tumor cells and then delivered into the acidic lysosomes through FA receptor-transduced endocytosis.

Next, the killing effect of NOSH@PEG-HCuSNPs was evaluated by CCK-8 assay on 4T1 cells. As shown in Fig. S16, although HCuSNPs alone had no obvious killing effect, single NOSH or NOSH@PEG-HCuSNPs showed obvious cell inhibition after administration, which means that NOSH and NOSH@PEG-HCuSNPs may promote 4T1 cell apoptosis by releasing NO and H_2_S, thereby significantly killing tumors. As shown in Fig. 3C, after laser irradiation, the cytotoxicity of HCuSNPs was significantly enhanced, and only 44 % of cells survived at high laser intensity, reflecting their excellent photothermal conversion properties. In contrast, NOSH@PEG-HCuSNPs had a more significant killing effect on tumor cells, and laser irradiation further amplified this effect. For example, a closed contrast found that only 21 % of tumor cells survived at a high laser intensity, while 26 % of cells survived at low laser intensity. These results indicated that NO and H_2_S gas played an important role in promoting the sensitivity of mPTT. Meanwhile, the live cell (green fluorescence channel)/dead cell (red fluorescence channel) co-staining method was also employed to verify the toxicity of NOSH@PEG-HCuSNPs toward 4T1 cells. As shown in Fig. 3D, a large number of dead cells appeared after irradiation of 200 μg mL^−1^ of NOSH@PEG-HCuSNPs with 0.5/1 W cm^−2^ of 808 nm laser for 10 min. The excellent killing effect of NOSH@PEG-HCuSNPs under 808 nm laser irradiation on 4T1 cells might be ascribed to FA receptor-mediated cellular uptake and an effective synergistic role between GT and mPTT. Considering that both H_2_S and NO can induce apoptosis through various pathways, it is necessary to clarify the ability of NOSH@PEG-HCuSNPs to release H_2_S and NO in cells. In detail, the H_2_S-specific fluorescent probe NP-N_3_ and NO-specific fluorescent probe Diaminofluorescein-FM diacetate (DAF-FM DA) were employed to evaluate the regulatory performance of intracellular H_2_S and NO after different pretreatments. CLSM results in Fig. 3E showed that the intracellular H_2_S and NO levels remained unchanged in the blank group and HCuSNPs-incubated cells with or without laser irradiation. Both NOSH and NOSH@PEG-HCuSNPs-treated cells showed the highly green fluorescence, indicating that there was a large amount of H_2_S and NO in 4T1 cells. A closed observation found that the fluorescence intensity of NOSH@PEG-HCuSNPs was changed upon the increase of the laser intensity, which not only directly confirmed that NOSH was well loaded in HCuSNPs, but also showed that the temperature change resulted from the laser intensity could amplify the hydrolysis and release of H_2_S and NO from NOSH@PEG-HCuSNPs (Figs. S17A and B). In addition, the photothermal process will also lead to excessive production of ROS. Therefore, the ROS probe 2′,7′-Dichlorodihydrofluorescein diacetate (DCFH-DA) was used to estimate the changes in the total intracellular ROS level in different pretreatment groups. As shown in Fig. S17C, CLSM results exhibited that the fluorescence intensity of HCuSNPs and NOSH@PEG-HCuSNPs under 808 nm laser irradiation was much higher than that of other groups. It was worth noting that the fluorescence intensity in groups with laser irradiation was stronger than those without laser irradiation, indicating that NOSH@PEG-HCuSNPs could generated a large amount of ROS while undergoing photothermal conversion, and the laser intensity could have an obvious effect on of ROS generation. Considering that a large amount of NO and ROS coexist in tumor cells, NO will be converted to ONOO^−^ [13], the ONOO^−^ detection kit was used to evaluate the ONOO^−^ level in 4T1 cells. As expected, 4T1 cells-treated with NOSH@PEG-HCuSNPs under 808 nm laser irradiation had a much higher ONOO^−^ levels compared other groups (Fig. S17D). Once again, the ONOO^−^ level in groups with laser irradiation was higher than those without laser irradiation. Therefore, the above-mentioned results roundly confirmed that NOSH@PEG-HCuSNPs could cascade to generate a large amount of H_2_S, NO, and ONOO^−^ under laser irradiation, which will provide a prerequisite for reinforcing ICD.

Some studies have revealed that high concentrations of H_2_S and NO interfere with the mitochondrial respiratory chain by downregulating COX IV to block the energy supply of tumor tissues. Inspired by this point, the membrane potential change of mitochondria in tumor cells-treated with different pretreatment groups were determined by the JC-1 assay. As shown in Fig. 3F, a closed contrast could be found in CLSM images that 4T1 cells-treated with the single NOSH had more mitochondrial membrane potential loss compared with the single HCuSNPs. What's more, in contrast with 4T1 cells-treated with other groups, 4T1 cells-treated with NOSH@PEG-HCuSNPs under 808 nm laser irradiation showed the largest reduction in JC-1 aggregates, the generation in JC-1 monomers, and the loss in mitochondrial membrane potential. Besides, mitochondrial dysfunction also resulted in the blockage of ATP production in 4T1 cells. In view of this, ATP, as the main energy source in cells, was quantified to explore the function of NOSH@PEG-HCuSNPs. As shown in Fig. S18, this group-induced by NOSH could reduce ATP generation. It is worth noting that the photothermal effect of HCuSNPs under laser irradiation also reduced ATP generation. This phenomenon might be due to the damage of endogenous biomolecules to the thermal effect. Based on the dual-acting of GT and mPTT, NOSH@PEG-HCuSNPs under 808 nm laser irradiation could reduceATP levels. As expected, ATP depletion was induced by the inhibition of the respiratory chain, indicating that COX IV levels significantly reduced in 4T1 cells-treated with NOSH@PEG-HCuSNPs under 808 nm laser irradiation (Fig. 3G). For downstream events, whether the overactivation of HSP 90 could be inhibited by energy depletion was also investigated. As shown in Fig. 3G, HCuSNPs-mediated photothermal heating increased the HSP 90 level in 4T cells. In contrast, H_2_S donors had an obvious effect on downregulating the expression of HSP 90, thereby reducing the level of HSP 90 in the combined treatment group to the level of the blank group. This downstream regulation of the energy supply by mitochondrial dysfunction suggested that H_2_S and NO were involved during the cell inhibition process, this result was further verified by the apoptosis kit Annexin V&PI. As shown in Figs. 3H and S19, although 4T1 cells-treated with the single NOSH induced the apoptosis. In sharp contrast, the combined therapeutic groups showed significant apoptosis. A closed contrast found that apoptosis in the same group with the high laser irradiation were much stronger than that with the low laser irradiation. Importantly, NOSH@PEG-HCuSNPs significantly reduced mitochondrial membrane potential and reduced ATP production, indicating that H_2_S triggers apoptosis by interfering with the mitochondrial respiratory chain as the main mechanism. The generation of ONOO⁻ indicates that the interaction between NO and ROS further amplifies the apoptotic effect. Combined with the results of the combination treatment group in Fig. 3, it shows that the synergistic effect of NO and H_2_S is crucial in tumor cell killing. Hence, the above experiments jointly confirmed that NOSH@PEG-HCuSNPs under 808 nm laser irradiation could achieve an obvious synergistic anti-tumor effect-mediated by GT and mPTT.

### 3.5 The associated mechanism of GT and mPTT in NOSH@PEG-HCuSNPs

**Fig. 4:**
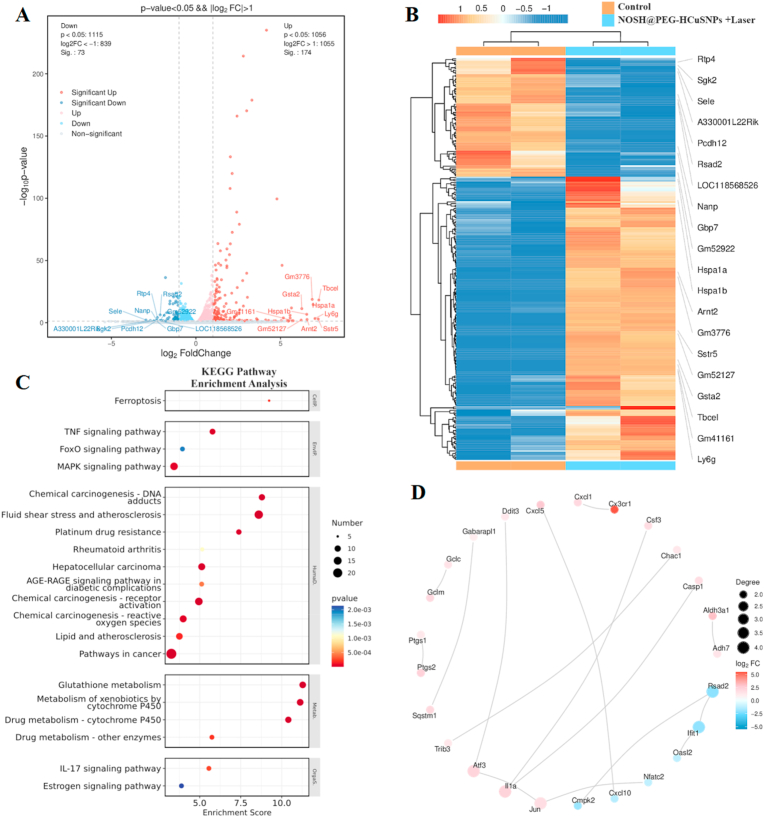
**The associated anti-tumor mechanism exploration of NOSH@PEG-HCuSNPs under 808 nm laser irradiation. (**A) Venn diagram of the differentially expressed genes (DEGs) between the blank control group and the therapeutic group (NOSH@PEG-HCuSNPs with 808 nm laser irradiation). (B) Heat map of apoptosis-related DEGs between the blank control group and the therapeutic group, red and green indicate significantly up-regulated genes and significantly down-regulated genes, with a fold change of ≥2.0 (or −2.0), *P* < 0.05. (C) KEGG pathway enrichment analysis of DEGs. (D) Protein-protein interaction relationship diagram related to signaling pathways. (For interpretation of the references to color in this figure legend, the reader is referred to the Web version of this article.)

In order to investigate the potential therapeutic mechanism of NOSH@PEG-HCuSNPs under 808 nm laser irradiation, the mRNA profiles of 4T1 cells treated with PBS (control group) and NOSH@PEG-HCuSNPs under 808 nm laser irradiation (therapeutic group) was analyzed by whole-genome RNA sequencing. The transcriptional profiles of *ca.* 15,000 genes in the experimental and control groups were explored and then 247 expression genes with the significant difference were screened out by the screening criteria (FC ≥ 2.0 (or - 2.0), *P* < 0.05). Compared with the cells in the control group, 174 genes were upregulated (marked as red dots) and 73 genes were downregulated (marked as blue dots) in 4T1 cells-treated with NOSH@PEG-HCuSNPs under 808 nm laser irradiation (see Figs. S20 and 4A). Besides, the genes with significant changes in the experimental group are listed in the heat map of the gene expression (Fig. 4B). Compared with the control group, the related apoptotic genes (Sstr5 and Arnt2) protection genes against heat damage (Hspa1a and Hspa1b), and immune response genes (Ly6g and Rsad2) significantly changed. In addition, GO gene enrichment showed that the upregulated genes were involved in apoptosis and oxidative damage protection, which was conducive to tumor regulation (see Fig. S21), while the downregulated genes were closely associated with immune regulation (Fig. S22). In order to better understand how gas signaling molecules in NOSH@PEG-HCuSNPs enhance the effect of PTT, KEGG pathway enrichment analysis was conducted (Fig. 4C), indicating that among the most significant cell pathways, multiple pathways such as TNF signaling pathway, FoxO signaling pathway, and MAPK signaling pathway were significantly enriched in 4T1 cells in the therapeutic group. Notably, previous studies have revealed that these pathways as the key signaling pathways can mediate cell apoptosis, anti-oxidative stress response, and immune response [[40], [41], [42]]. Meanwhile, the protein-protein interaction network was further constructed (Fig. 4D). The key gene ATF3 (activating transcription factor 3) in this network was a stress-induced transcription factor that was involved in the occurrence, development, therapy, and prognosis of tumors. By participating in multiple signaling pathways, ATF3 had an obvious proliferation on breast cancer cells and played a suppressor role in breast cancer. Therefore, the above results revealed that the therapeutic group might take advantage of activating multiple signaling pathways under the synergistic effects of GT and mPTT, thereby promoting the apoptosis of cancer cells.

### 3.6 *In vivo* antitumor evaluation

**Fig. 5:**
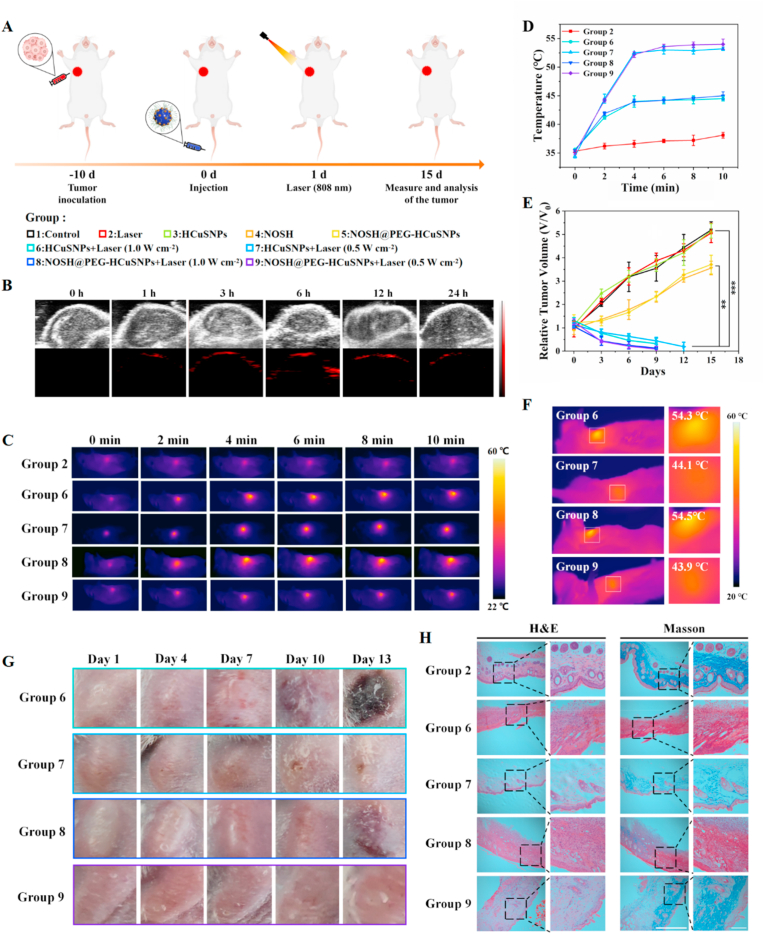
Therapeutic study and wound analysis of NOSH@PEG-HCuSNPs in 4T1 tumor-bearing mice. (A) Schematic diagram of combined treatment of tumor-bearing mouse model with NOSH@PEG-HCuSNPs. (B) Photoacoustic imaging of tumor sites of 4T1 tumor-bearing mice intravenously injected with NOSH@PEG-HCuSNPs at different time points. (C) Thermal images of tumor areas of tumor-bearing mice in different groups within 10 min of laser irradiation. (D) Time-dependent curves of the temperature of tumor areas of tumor-bearing mice in different groups under the laser. (E) Tumor volume change curves of each group after different treatments. (F) Thermal imaging during PTT of different treatment groups. (G) Macroscopic observation of laser-irradiated skin sites of mice in each group on days 1, 4, 7, 10, and 13. (H) Corresponding H&E and Masson-stained skin images and magnified images of tumor irradiated sites of each group after treatment. Scale bar = 200 μm; All experiments were performed at least three times. Each value represents the mean ± SD (n = 3). Significant differences were calculated by one-way ANOVA (∗∗∗P < 0.001, ∗∗P < 0.01, or ∗P < 0.05).

Inspired by the anti-tumor efficacy of the above cells, in order to evaluate the therapeutic effect of NOSH@PEG-HCuSNPs and further explore the potential feasibility of NOSH@PEG-HCuSNPs *in vivo* against tumors, a solid tumor model of 4T1 cells in the right axilla of Balb/c mice. Compared with some more complex tumor models, this tumor-bearing mouse model has better universality. As shown in Fig. 5A. Balb/c tumor-bearing mice transplanted with 4T1 tumor cells were randomly divided into groups to systematically verify the *in vivo* anti-cancer effect of combined therapy. Next, ICG-labeled NOSH@PEG-HCuSNPs were intravenously injected into mice, and their dynamic changes in mice were tracked by *in vivo* fluorescence imaging technology to determine the appropriate time for laser irradiation to activate PTT. As shown in Fig. S23, NOSH@PEG-HCuSNPs gradually accumulated in the tumor area of mice after intravenous injection. The accumulated NOSH@PEG-HCuSNPs reached a peak at *ca.* 6 h after injection and then gradually weakened. Since NOSH@PEG-HCuSNPs have excellent photothermal conversion ability, they can be directly used as photoacoustic (PA) imaging agents to further evaluate their aggregation at the tumor site. The PA signals at the tumor site were monitored after imaging of tumor-bearing mice injected intravenously with NOSH@PEG-HCuSNPs at different time intervals. As shown in Fig. 5B and Fig. S24, the PA signal in the tumor area increased in a time-dependent manner, and the PA signal at the tumor site of the mouse reached the maximum 6 h after intravenous injection, which was consistent with *in vivo* fluorescence imaging. Next, the temperature changes in the tumor area of Balb/c tumor-bearing mice after laser irradiation were recorded with a thermal imager to evaluate the photothermal effect of the material *in vivo* after intravenous administration. As shown in Fig. 5C and D, after 808 nm laser irradiation (0.5 W cm^−2^) for 10 min, the temperature at the tumor site of the PBS-administered group was almost unchanged, while the temperature of the drug-administered group significantly increased. It is noteworthy that after 808 nm laser irradiation for 10 min, the temperature of the HCuSNPs and NOSH@PEG-HCuSNPs groups increased to *ca.* 45 °C at low laser intensity (0.5 W cm^−2^), while it increased to *ca.* 54 °C at high laser intensity (1 W cm^−2^). In short, this above results well revealed that light-to-heat conversion rate of NOSH@PEG-HCuSNPs is high enough to meet the therapeutic requirements.

To verify the biocompatibility and clinical application potential of NOSH@PEG-HCuSNPs, we comprehensively evaluated its potential toxicity *in vivo* on Balb/c mice. As shown in Fig. S25, the weight loss of mice during the entire treatment period was negligible, indirectly indicating that NOSH@PEG-HCuSNPs had obvious therapeutic biosafety. After intravenous injection of therapeutic doses of PBS, HCuSNPs, and NOSH@PEG-HCuSNPs for 14 days, no obvious histopathological lesions were found in the hematoxylin-eosin (H&E) staining images of the main tissues and organs (heart, liver, spleen, lung, and kidney) of Balb/c mice (Fig. S26). In addition, the results of routine blood analysis also showed that NOSH@PEG-HCuSNPs had almost no toxicity to Balb/c mice during the entire observation period (Fig. S27).

Subsequently, the therapeutic effect of NOSH@PEG-HCuSNPs was evaluated. Six hours after the tail vein injection, the tumor site was irradiated with an 808 nm laser for 10 min. The mice received five rounds of treatment during the treatment period and were sacrificed on the last day. To reveal the effects of different treatment operation temperatures on the efficacy and corresponding side effects of PTT, HCuSNPs, and NOSH@PEG-HCuSNPs were operated with two laser intensities (0.5 W cm^−2^ or 1 W cm^−2^), respectively. As for the results, as shown in Fig. 5E, the tumor growth of the single PBS group, the single laser irradiation group, and the single HCuSNPs group were not inhibited, but on the contrary, the size of the tumor was several times that before treatment. However, the single administration of NOSH and NOSH@PEG-HCuSNPs showed a limited inhibitory effect on tumor growth, which may be attributed to the anti-cancer effect caused by the release of a certain amount of NO and H_2_S in the tumor by NOSH *in vivo*, which promotes the apoptosis of cancer cells. Most importantly, although the photothermal effect of HCuSNPs under 808 nm laser irradiation more significantly inhibited tumor growth with the increase of laser intensity, NOSH@PEG-HCuSNPs showed a better therapeutic effect with the further support of NO and H_2_S released by NOSH, that is, the mPTT effect of NOSH@PEG-HCuSNPs under low laser intensity was consistent with the PTT effect under high power, and both completely eradicated the tumor on the 9th day after treatment. As shown in Fig. S28, the tumor images collected during the treatment further showed that the effect of NOSH had a strong enhancing effect on the therapeutic effect of NOSH@PEG-HCuSNPs.

Finally, to clarify the therapeutic mechanism of NOSH@PEG-HCuSNPs, we performed histological analysis, and further performed H&E, terminal deoxynucleotidyl transferase-mediated deoxyuridine triphosphate nick end labeling (TUNEL) staining, and Ki67 antibody staining to observe the inhibitory effect of the treatment on tumor cells and further evaluate the efficacy of each treatment. As shown in Fig. S29, the H&E microscopic image intuitively shows that although cell necrosis occurred in the single NOSH, NOSH@PEG-HCuSNPs, and HCuSNPs + laser groups, the degree of cell necrosis in the NOSH@PEG-HCuSNPs + laser group was significantly higher than that in the other treatment groups. In addition, the results of TUNEL staining to detect tumor cell apoptosis showed that more TUNEL-positive cells appeared in the NOSH@PEG-HCuSNPs + laser group, and the tumor cell nuclei after combined treatment were loosely arranged, indicating that it has outstanding antitumor activity and induced tumor cells with a high apoptosis rate. This result, together with the downregulated expression of the cell proliferation indicator Ki67, further supports the enhanced efficacy of PTT caused by H_2_S and NO released by NOSH. It is worth noting that the laser intensity has no significant effect on the anti-tumor therapeutic effect of NOSH@PEG-HCuSNPs. Therefore, combined with the above experimental results, we believe that NOSH@PEG-HCuSNPs not only maintain excellent photothermal conversion and photothermal ablation capabilities *in vivo* under laser irradiation, but also the H_2_S and NO that can be continuously released in the tissue during the treatment process enhance the photothermal therapeutic effect, enabling it to achieve the best anti-tumor therapeutic effect under the mPTT of low-power laser.

### 3.7 Characterization of skin tissue integrity during NOSH@PEG-HCuSNPs treatment

As shown in Fig. 5F, during the treatment, the operating temperature of HCuSNPs and NOSH@PEG-HCuSNPs under high-power laser was about 54 °C, while the operating temperature of HCuSNPs and NOSH@PEG-HCuSNPs under low-power laser was about 44.0 °C. Through naked eye observation of the laser-irradiated skin area, it can be seen from Fig. 5G that after the second treatment, the skin and surrounding tissues of the high laser intensity groups were found to be red and swollen, gradually developing into ulcerative lesions, while the damage to the 808 nm laser irradiation position was not observed until the end of the treatment process under low laser intensity. The above confirms that the treatment process induced by a low-energy laser (0.5 W cm^−2^) can cause negligible damage to normal tissues. Among them, the high-power laser group of NOSH@PEG-HCuSNPs showed obvious skin damage after the fourth treatment, while the high-power laser group of HCuSNPs showed visible damage to the naked eye after the second treatment. To clarify the mechanism, we further investigated the integrity of the skin tissue at the 808 nm laser irradiation site after treatment by tissue sections. As shown in Fig. 5H–H&E staining showed skin tissue damage under a high laser, with obvious inflammation and tissue hardening. This damage to the epidermal tissue not only increases the risk of microbial infection during treatment, but also affects the penetration effect of the laser and impairs the photothermal conversion performance of NOSH@PEG-HCuSNPs in the tumor area. Considering that collagen deposition is an important indicator of granulation tissue formation and re-epithelialization, we also evaluated the effect of treatment on collagen in the skin by Masson staining. In high laser treatment, only a few collagen fibers were found in the epidermis of the wound bed, indicating that the wound healing process was hindered. In contrast, the skin maintained good integrity during low laser treatment, and the damage was negligible. However, it is worth noting that, similar to the results of the eye observation mentioned above, at the same laser intensity, the skin sections of the NOSH@PEG-HCuSNPs laser group had fewer inflammation areas, more collagen, and milder damage. Therefore, this further demonstrates the superiority of PTT combined with gas molecules, and the combined therapy demonstrated by NOSH@PEG-HCuSNPs provides a sustainable and reproducible mPTT process for tumor treatment.

### 3.8 NOSH@PEG-HCuSNPs reverse the immunosuppressive microenvironment

**Fig. 6:**
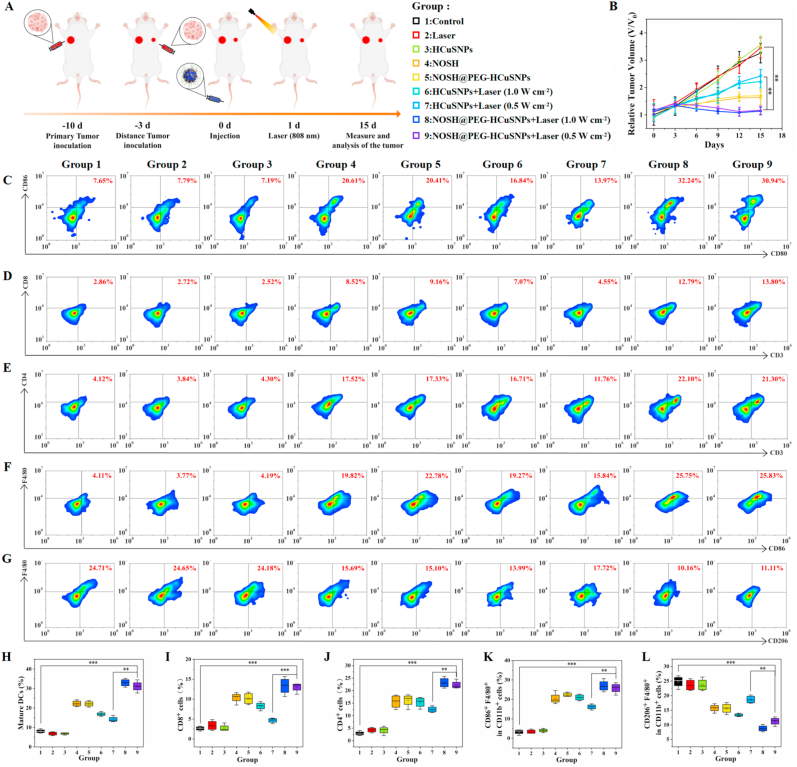
Anticancer effect of NOSH@PEG-HCuSNPs-mediated combined therapy on distant tumors. (A) Schematic diagram of NOSH@PEG-HCuSNPs inhibiting the growth of primary and distant tumors. (B) Curves of changes in distant tumor volume in each group after different treatments. (C) Representative flow cytometry analysis of maturation DCs (CD80^+^ CD86^+^ gated on CD11c^+^) in tumor-draining lymph nodes. (D) Representative flow cytometry analysis of CTLs (CD8^+^ CD3^+^) in the treated tumor. (E) Representative flow cytometry analysis of CD4 Helper T Cells (CD4^+^ CD3^+^) in the treated tumor. (F) Representative flow cytometry analysis of M1 macrophages (CD206^+^ F4/80^+^ gated on CD11b^+^) in the treated tumor. (G)Representative flow cytometry analysis of M2 macrophages (CD86^+^ F4/80^+^ gated on CD11b^+^) in treated tumor. (H)Percentage of maturation DCs (CD80^+^ CD86^+^ gated on CD11c^+^) in tumor-draining lymph nodes. (I)Percentage of CTLs (CD8^+^ CD3^+^) in the treated tumor. (J)Percentage of CD4 Helper T Cells (CD4^+^ CD3^+^) in the treated tumor. (K)Percentage of M1 macrophages (CD206^+^ F4/80^+^ gated on CD11b^+^) in the treated tumor. (L)Percentage of M2 macrophages (CD86^+^ F4/80^+^ gated on CD11b^+^) in the treated tumor. All experiments were performed at least three times. Each value represents mean ± SD (n = 3). Significant differences were calculated by one-way analysis of variance (∗∗∗P < 0.001, ∗∗P < 0.01, or ∗P < 0.05).

According to previous reports, local tumor hyperthermia can stimulate systemic anti-tumor immunity to attack distal tumors while destroying the primary tumor [43,44]. In addition, gaseous signaling molecules can also reverse the immune microenvironment by participating in multiple signaling pathways. Therefore, encouraged by the significant therapeutic effect of the primary tumor model, as shown in Fig. 6A, we established a dual tumor model to verify the inhibitory effect of the combined treatment regimen on distal tumors to evaluate the potential of NOSH@PEG-HCuSNPs to prevent tumor recurrence and metastasis. When the volume of the primary tumor reached approximately 50 mm^3^, 4T1 cells were subcutaneously inoculated as distal tumors on the contralateral side of the mouse. The mice were then randomly divided into groups and treated according to the indicated formula. The volume of the primary tumor and the distal tumor was recorded every other day. The tumor growth curve of the distal tumor (Fig. 6B) showed that the PBS group, laser group and HCuSNPs group had no inhibitory effect on the size of the distal tumor, and the size continued to increase over time, approaching three times the size at the beginning of the experiment on 15 days. However, compared with the HCuSNPs group, under the action of laser, HCuSNPs showed a certain inhibitory effect on the distal tumor while thermally ablating the primary tumor (Fig. S30), which was attributed to the ICD effect produced during photothermal ablation of the tumor. In addition, due to the effect of NOSH releasing gas signal molecules, the NOSH and NOSH@PEG-HCuSNPs single-administration groups showed obvious inhibitory effects on the distal tumor, which only increased by about 1.5 times compared with the beginning of the experiment. When NOSH@PEG-HCuSNPs were irradiated by laser after administration, the distal tumor of the combined treatment group had almost no growth compared with other groups, showing a significant inhibitory effect on the distal tumor. It is worth noting that under different laser intensities, the effect of ablation of primary tumors gradually increased with the intensity of the laser, but the effect of laser intensity on the inhibition of distal tumors was not significant, which might be ascribed to the limited ICD effect caused by photothermal treatment. At the same time, the tumor images collected during the treatment process corroborated the above experimental results (Fig. S32). In addition, there was no significant fluctuation in the weight of mice during the treatment (Fig. S32). These results all indicate that the ICD effect produced by NOSH@PEG-HCuSNPs under laser treatment and the synergistic effect of gas signaling molecules produce a strong systemic anti-tumor activity.

In order to clarify that the good therapeutic effect is related to the activation of anti-tumor immune response mediated by NOSH@PEG-HCuSNPs, we collected tumors and their tumor-draining lymph nodes from mice and analyzed the proportion of immune-related cells by flow cytometry. Damage associated molecular patterns from dead cells serve as "danger signals" of the host immune system. They induce DC maturation to activate cytotoxic T lymphocytes (CTLs)-mediated anti-tumor efficacy and inhibit tumor recurrence and metastasis [9,45]. On this basis, we used flow cytometry to evaluate the maturation status of DC cells (CD11c^+^CD80^+^CD86^+^) in lymph nodes. Excitingly, as shown in Fig. 6C and H, compared with other groups, the cells in the combined treatment group of NOSH@PEG-HCuSNPs plus laser showed the most significant DC cell maturation ratio. This suggests that the destruction of primary tumors by NOSH@PEG-HCuSNPs-mediated combined therapy may expose more tumor epitopes, and the resulting ICD caused by gaseous signaling molecules and PTT stimulates DC cells to initiate a powerful antigen presentation function to activate T cells.

Therefore, we simultaneously used flow cytometry to analyze CD4^+^ T cells and CD8^+^ T cells in the tumor site to understand the antitumor activity of T lymphocytes after NOSH@PEG-HCuSNPs combined treatment. On the one hand, CTLs (CD3^+^CD8^+^) can trigger specific oncolysis of tumor cells by releasing cytotoxins [46]. We further examined the CTLs of tumor tissue after different administrations and treatments, as shown in Fig. 6D and I. The results showed that compared with other groups, the NOSH@PEG-HCuSNPs group under laser treatment significantly enhanced the infiltration of CTLs, and the CD8^+^ cells that released cytotoxins to kill tumor cells increased significantly. On the other hand, after NOSH@PEG-HCuSNPs mediated mPTT, the percentage of CD4^+^ T cells (also known as helper T lymphocytes) can maintain and enhance potential immune function [47], which is 5 times that of the single PBS group and 2 times that of the HCuSNPs mild laser treatment group (Fig. 6E and J).

In addition, as an important immunosuppressive cell, the reverse polarization of M2 macrophages is particularly important in anti-tumor immunity. It is known that the functional phenotype of macrophages can be adaptively readjusted according to changes in the environment [48]. The production of ONOO^−^ in tumor tissues caused by the combined treatment of NOSH@PEG-HCuSNPs can polarize M2 macrophages into M1 macrophages. Therefore, we carefully examined the phenotypic changes of macrophages in tumors. As shown in Fig. 6F and K, we found that the proportion of M1 macrophages (CD11b^+^F4/80^+^CD86^+^) in the combined treatment group of NOSH@PEG-HCuSNPs was significantly increased. At the same time, there was a trend opposite to the results of M1 macrophages, and the percentage of M2 macrophages (CD11b^+^F4/80^+^CD206^+^) after combined treatment was significantly reduced (Fig. 6G and L). The above results further suggest that ONOO^−^ produced by the bidirectional enhancement strategy mediated by the combined treatment of NOSH@PEG-HCuSNPs can significantly accelerate the polarization of macrophages from M2 to M1 in tumors, leading to the reversal of TME immunosuppression and promoting the recruitment of more CTLs to tumor tissues.

**Fig. 7:**
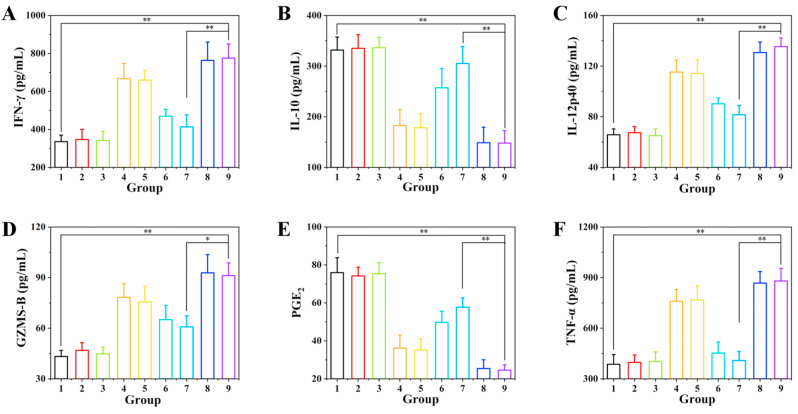
The differences in the levels of cytokines (IFN-γ (A), IL-10 (B), IL-12p40 (C), GZMS-B (D),PGE_2_ (E) and TNF-α (F)) in the serum supernatant of each group of mice were detected by ELISA after different treatments. All experiments were performed at least three times. Each value represents mean ± SD (n = 3). Significant differences were calculated by one-way analysis of variance (∗∗∗P < 0.001, ∗∗P < 0.01, or ∗P < 0.05).

To further confirm the anti-tumor immune ability of the NOSH@PEG-HCuSNPs-mediated bidirectional enhancement strategy, we used an ELISA kit to evaluate the levels of immune-related cytokines in the blood supernatant of mice after different treatments. The results are shown in Fig. 7. Compared with other groups, interferon γ (IFN-γ), which plays a decisive role in the differentiation of M1 macrophages [49], was significantly enhanced after NOSH@PEG-HCuSNPs-mediated combined treatment. At the same time, the cytokines TNF-α and IL-12, which are mainly secreted by M1 macrophages, were significantly increased [50,51] and the cytokine (IL-10) mainly secreted by M2 macrophages as a multifunctional negative regulator [52], was significantly downregulated after combined treatment. In addition, granzyme B (GZMS-B) [53], which plays a role in killing tumor cells in CTLs, and prostaglandin E2 (PGE_2_) [54], which helps tumor cells escape the immune system, were significantly increased and decreased in the blood supernatant of mice after NOSH@PEG-HCuSNPs-mediated combined treatment, respectively. H_2_S and NO play distinct yet complementary roles in enhancing mPTT efficacy. H_2_S inhibits HSP 90, reducing tumor thermotolerance and sensitizing cells to hyperthermia, especially under mild photothermal conditions (40–45 °C). It also protects normal tissues by maintaining redox balance and mitigating ROS-induced damage, improving treatment safety. NO amplifies oxidative damage by reacting with ROS to form ONOO⁻, promoting apoptosis, while also modulating the tumor immune microenvironment by enhancing ICD and boosting anti-tumor immunity. The laser-triggered dual release of H_2_S and NO ensures precise spatiotemporal synergy, enhancing cytotoxicity, targeting key pathways, and improving therapeutic outcomes. Obviously, this combination therapy has unique advantages over existing photothermal therapy, photodynamic therapy, and new tumor treatment methods [55,56]. After cascade synergistic treatment, the closely related specific immune response triggers a powerful low-damage anti-tumor effect, while reversing the immunosuppressive TME and synergistically inhibiting tumor metastasis and recurrence.

## 4 Discussion

In summary, we developed a NIR-triggered programmable nanomotor NOSH@PEG-HCuSNPs to activate sufficient immune response to enhance the non-invasive mPTT anti-tumor efficacy. By loading NOSH, a gas signaling molecule donor that releases H_2_S and NO on demand, onto hollow mesoporous copper sulfide nanoparticles with good photothermal conversion efficiency, the simultaneous and co-location delivery of NOSH and HCuSNPs was effectively achieved, thereby exerting the synergistic effect of GT and mPTT. Based on the effective sensitization of the dual-release gas donor NOSH, tumor cell apoptosis was promoted on the one hand, and HSP 90 expression was reduced on the other hand by synergistically inducing mitochondrial dysfunction. Thus, a completely non-invasive treatment process was observed without affecting the efficacy, greatly improving the side effects associated with PTT. The strategy of significantly reversing the tumor immune microenvironment by enhancing ICD through the cascade reaction of mPTT and GT effectively increased the proportion of mature DCs, promoted the polarization of macrophages to the M1 phenotype, activated CTLs against tumors, sensitized the systemic immune response system, and prevented the growth of distant tumors. The strategy of dual GT sensitization mPTT in this study not only provides a low-invasive method for thermal ablation of primary tumors, but also solves the problem of postoperative tumor recurrence and metastasis. It is expected to become a universal solution to improve the efficacy of mPTT and can serve as an important example of innovation in the field of tumor treatment in the future.

## CRediT authorship contribution statement

**Jinlong Zhang:** Writing – original draft, Visualization, Conceptualization. **Quan Jing:** Data curation. **Longlong Yuan:** Formal analysis. **Xianhui Zhou:** Formal analysis. **Duolong Di:** Funding acquisition. **Jinyao Li:** Investigation. **Dong Pei:** Resources. **Zhongxiong Fan:** Writing – review & editing, Supervision. **Jun Hai:** Visualization, Validation, Funding acquisition.

## Funding

This work was supported by the Science and Technology program of Gansu Province (24JRRA063), the Distinguished Young Researcher Program of Lanzhou Institute of Chemical Physics (E304A8SY), the 10.13039/501100001809National Natural Science Foundation of China (32360185), Key R&D Program of Yunnan Province (No. 202203AD150003), Yunnan Province Major Scientific and Technological Project (No. 202302AE090007 & 202402AA310034), the Key research and development program in Xinjiang Uygur Autonomous Region (2023B02030-1), Tianshan Talent Training Program (2023TSYCLJ0043), and the Qingdao Municipal Bureau of Science and Technology (24-1-4-xxgg-17-nsh), the Natural Science Foundation of Xinjiang Uygur Autonomous Region（2022D01D15).

## Declaration of competing interest

The authors declare that they have no known competing financial interests or personal relationships that could have appeared to influence the work reported in this paper.

## Data Availability

Data will be made available on request.
