# Supplementary material for: NIR-triggered programmable nanomotor with H2S and NO generation for cascading oncotherapy by three-pronged reinforcing ICD
Source: Mater Today Bio. 2025 Feb 3;31:101540. doi: 10.1016/j.mtbio.2025.101540 (PMC11848782; doi:10.1016/j.mtbio.2025.101540)
Supplement: Multimedia component 1 [file mmc1.docx]

***Supporting Information***

**NIR-Triggered Programmable Nanomotor with H_2_S and NO Generation for Cascading Oncotherapy by Three-Pronged Reinforcing ICD**

Jinlong Zhang^1^, Quan Jing^1,3^, Longlong Yuan^2^, Xianhui Zhou^4^, Duolong Di^1^, Jinyao Li^2,^ *, Dong Pei^1,^ *, Zhongxiong Fan^2,^ *, Jun Hai^1,^ *

^1^ CAS Key Laboratory of Chemistry of Northwestern Plant Resources and Key Laboratory of Natural Medicine of Gansu Province, Lanzhou Institute of Chemical Physics, Chinese Academy of Sciences, Lanzhou 730000, Gansu, China

^2^ School of Pharmaceutical Sciences, Institute of Materia Medica, Xinjiang University, Urumqi 830017, China

^3^ University of Chinese Academy of Sciences, Beijing 100049, China

^4^ Department of Cardiac Pacing and Electrophysiology, The First Affiliated Hospital of Xinjiang Medical University, Urumqi, 830054, China

* Correspondence: Dr. Jinyao Li, E-mail: ljyxju@xju.edu.cn; Dr. Dong Pei, E-mail: dongpei@licp.cas.cn; Dr. Zhongxiong Fan, E-mail: [fanzhongxiong@xju.edu](mailto:fanzhongxiong@xju.edu).cn; Dr. Jun Hai, E-mail: haijun@licp.cas.cn


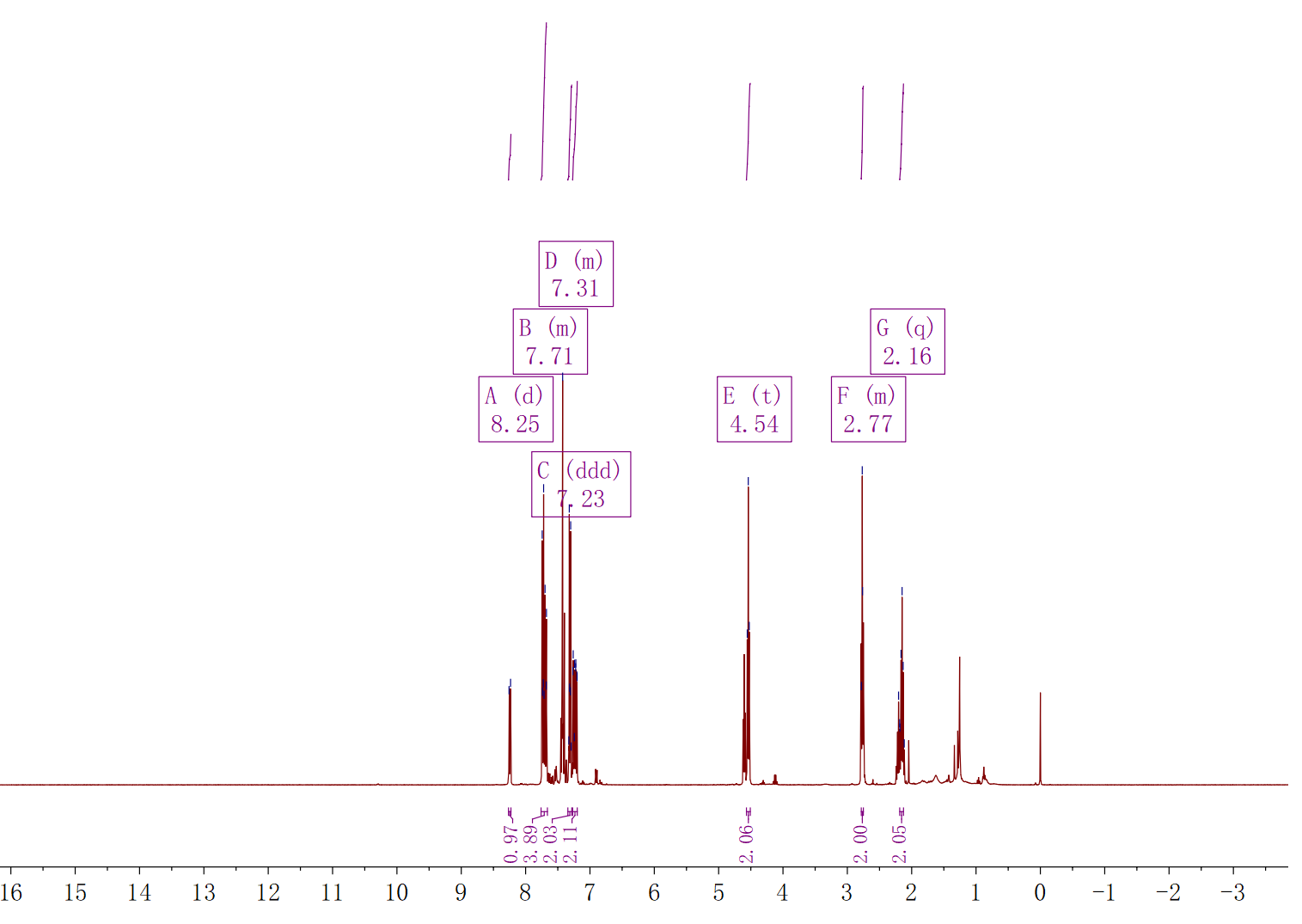


**Figure S1** ^1^H NMR of Compound NOSH


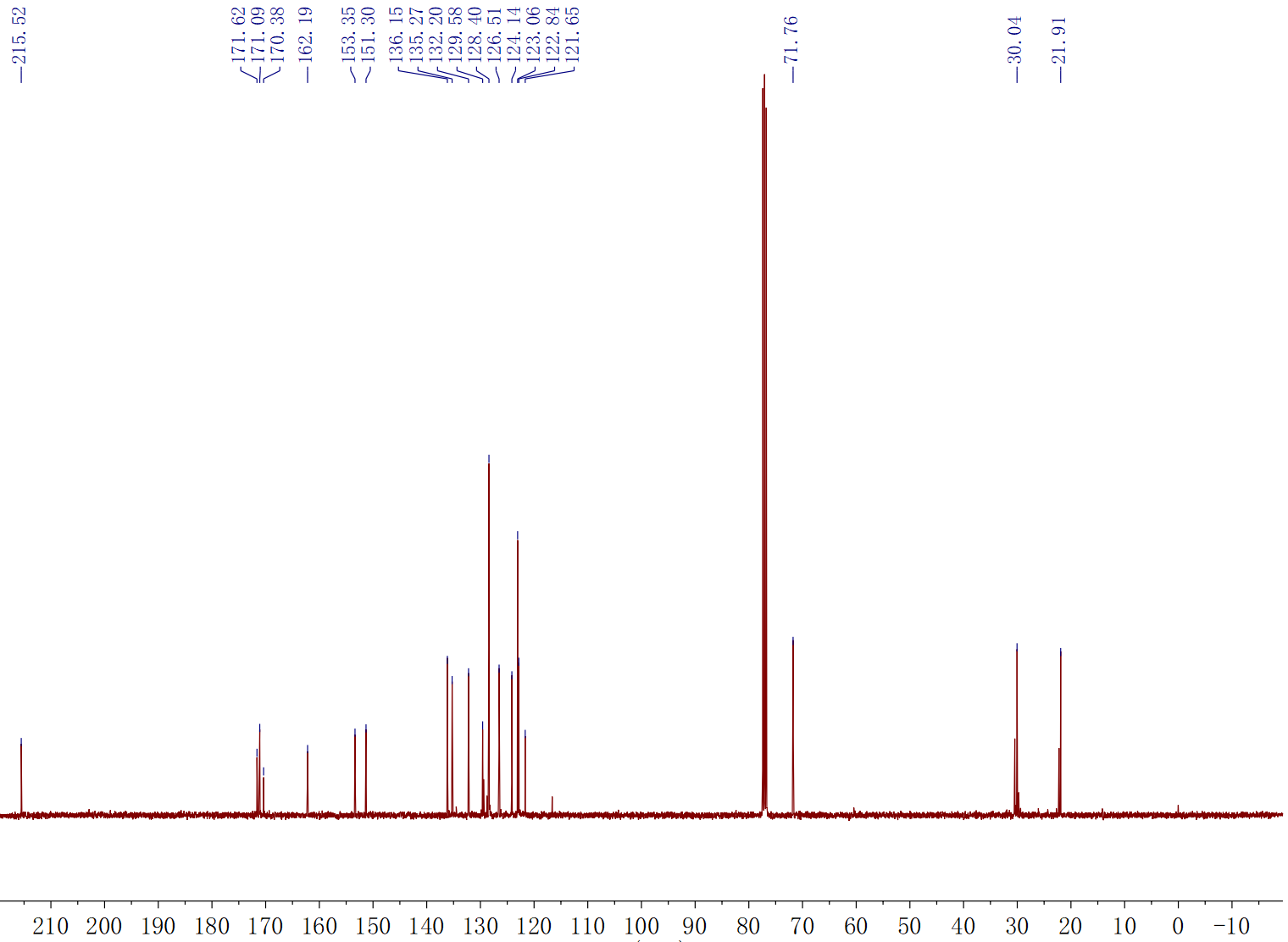


**Figure S2** ^13^C NMR of Compound NOSH


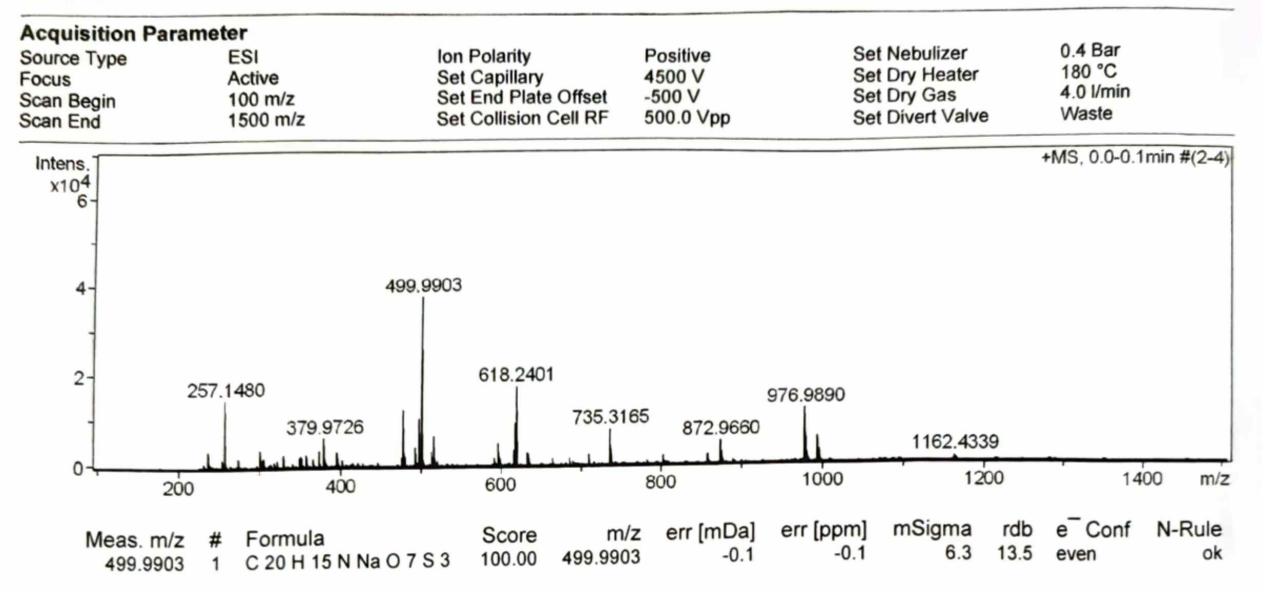


**Figure S3** High-resolution mass spectrometry of Compound NOSH


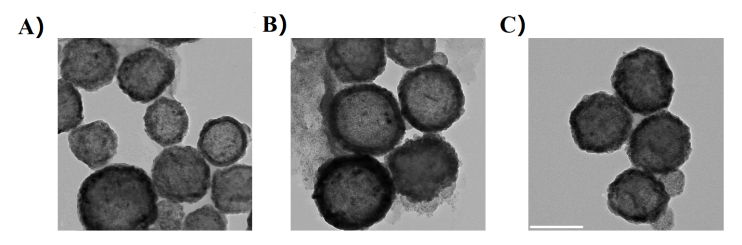


**Figure S4** TEM images of (A) HCuSNP, (B) NOSH@HCuSNPs and (C) [NOSH@PEG-HCuSNPs.](mailto:NOSH@PEG-HCuSNPs._x0005_)





**Figure S5** XRD spectra of HCuSNPs, NOSH@HCuSNPs and NOSH@PEG-HCuSNPs


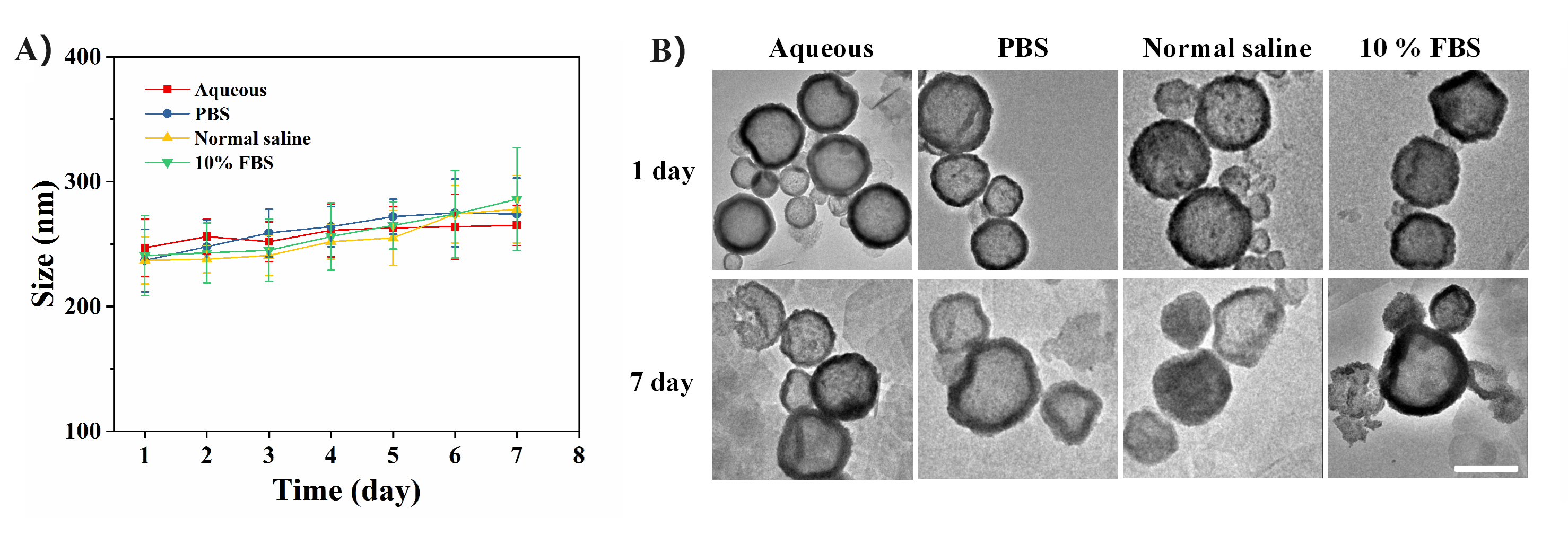


**Figure S6** Size changes and TEM images of NOSH@PEG-HCuSNP in Aqueous, PBS, Normal saline, and 10% FBS 1640. Each value represents the mean ± SD (n = 3). Scale bar (200 nm)


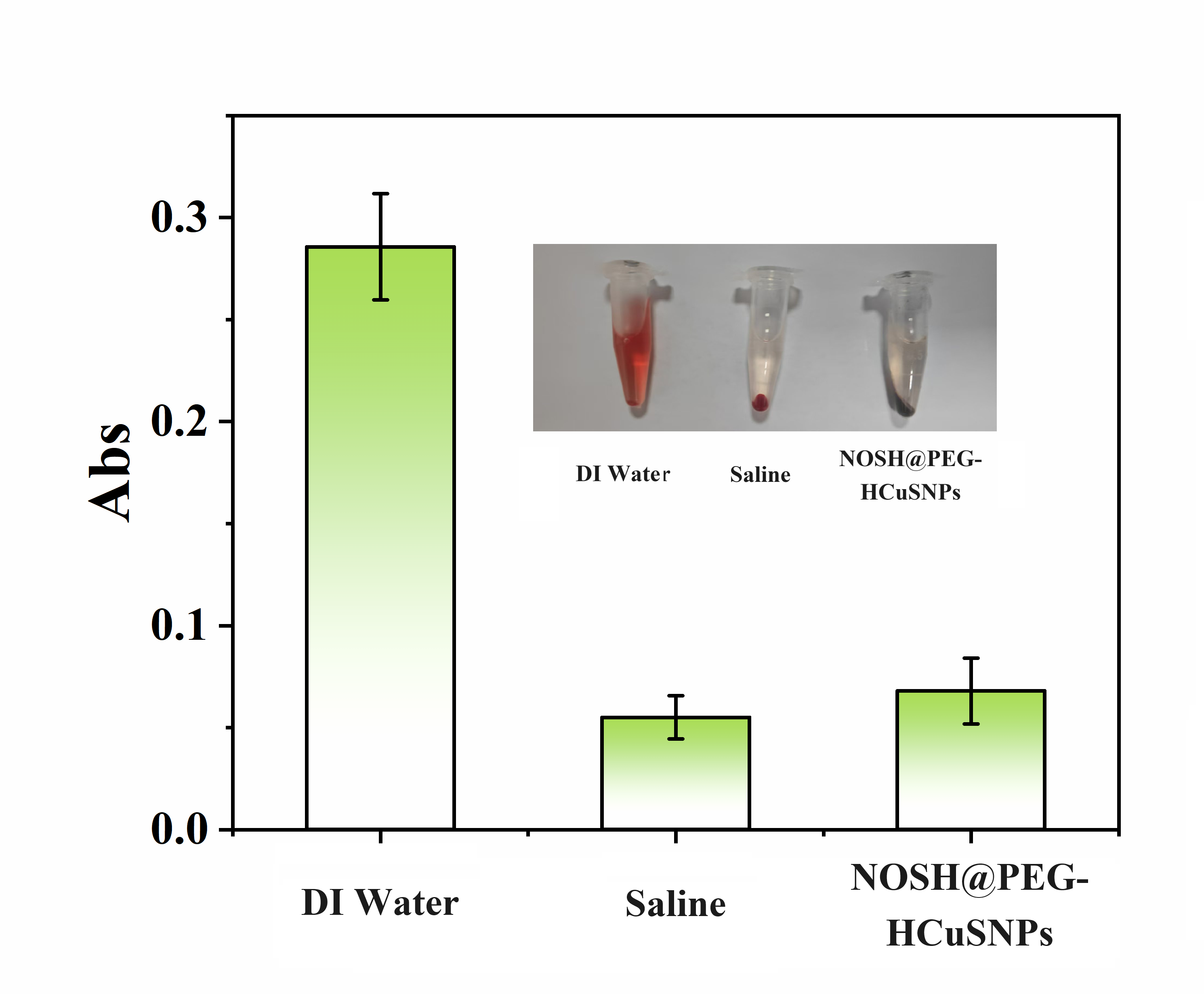


**Figure S7** Hemolytic effect of NOSH@PEG-HCuSNPs. DI water was used as the positive control and PBS was used as the negative control.





**Figure S8** UV-visible absorption spectrum of NOSH@PEG-HCuSNPs


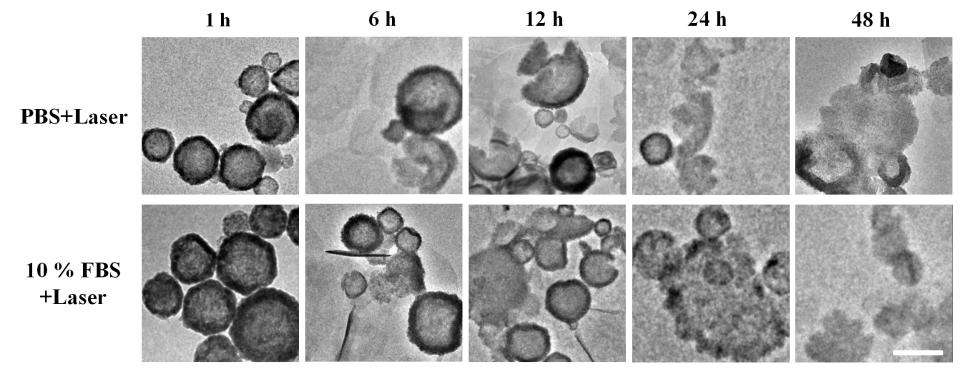


**Figure S9** TEM images of cells in PBS solution and 10% FBS-containing 1640 medium after different incubation times under laser irradiation.


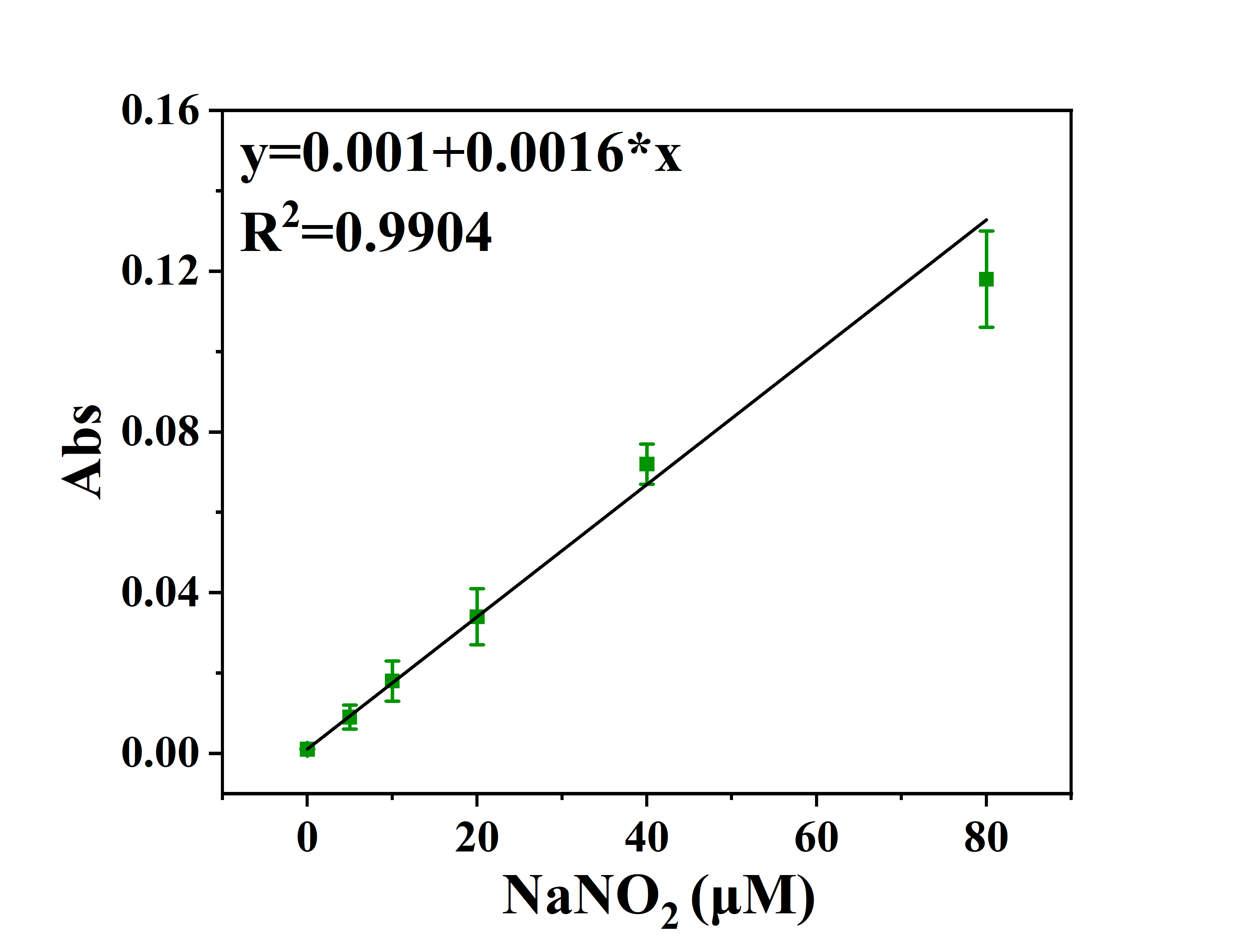


**Figure S10** Standard curve of the absorption of Griess reagent in response to NaNO_2_ at 540 nm.


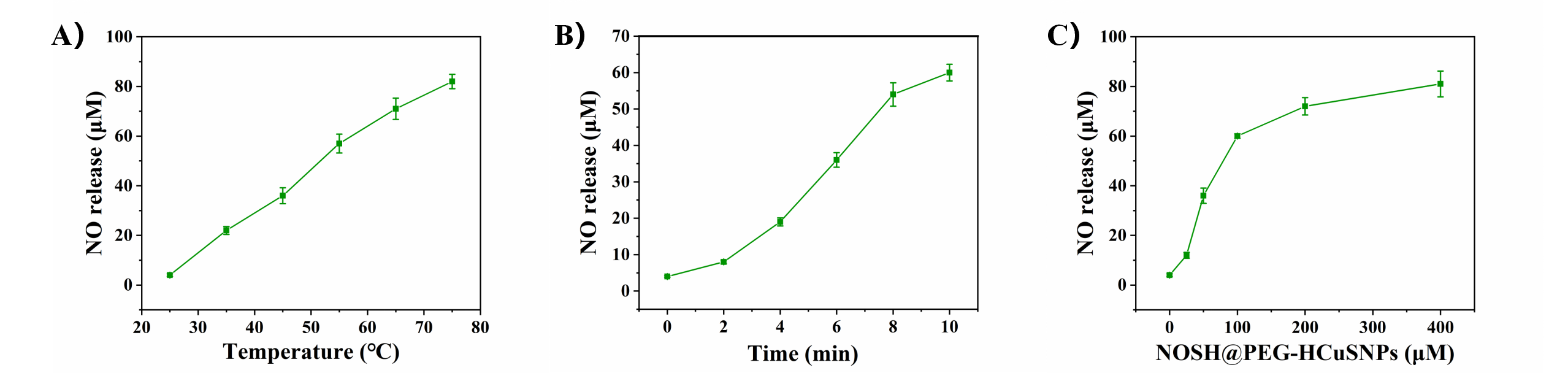


**Figure S11** The amount of NO released by NOSH@PEG-HCuSNPs at different temperatures (A), different times (B) and different concentrations (C).

**Figure S12 Chemical structure of NP-N_3_ and its reaction mechanism for detecting H_2_S**


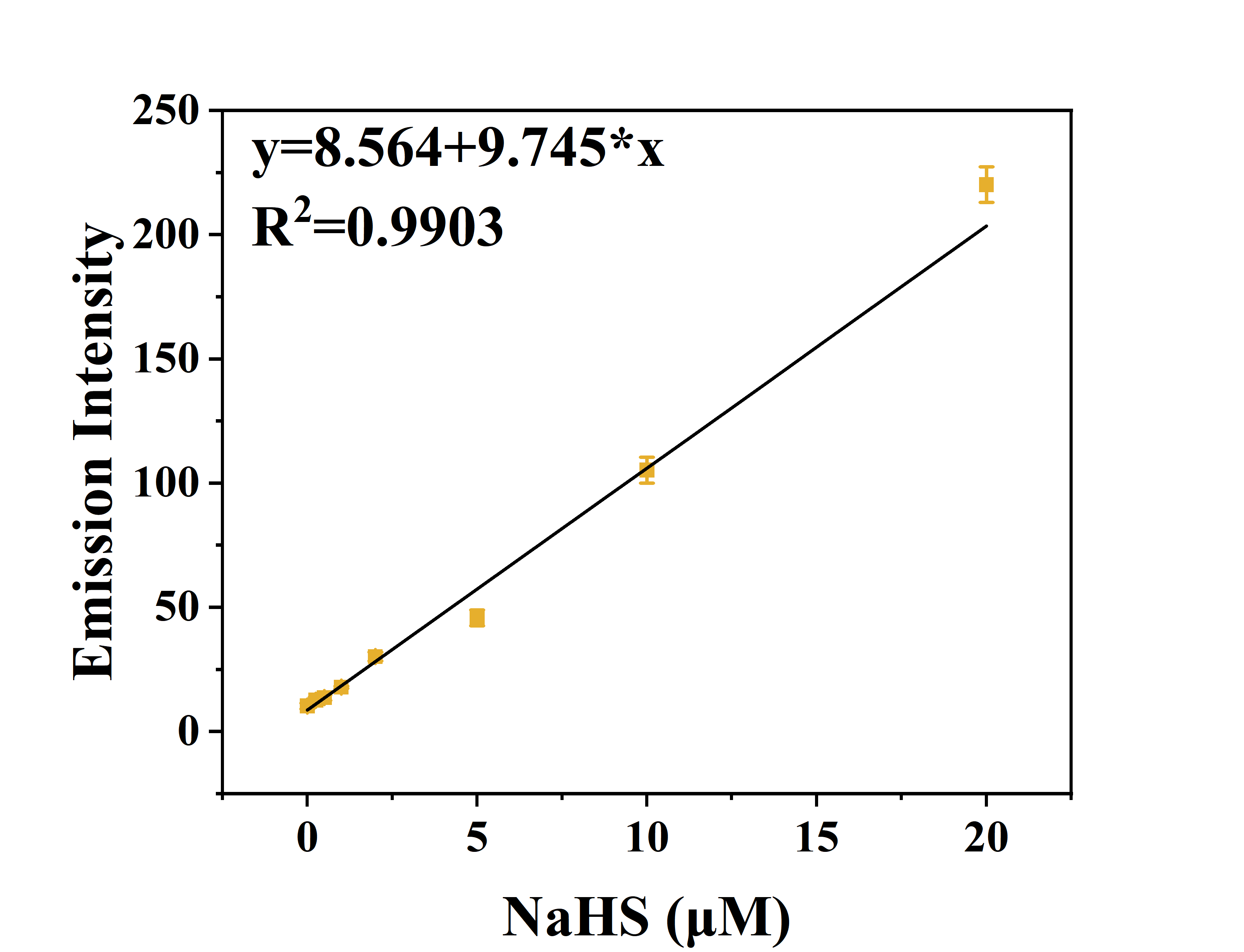


**Figure S13** The emission standard curve of probe NP-N_3_ in response to NaHS at 520 nm.


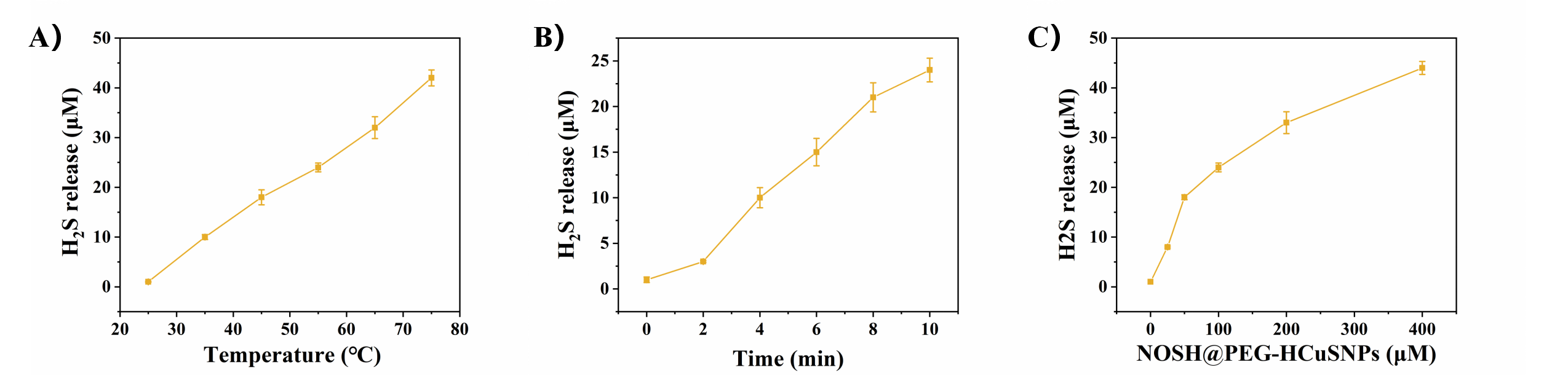


**Figure S14** The amount of H_2_S released by NOSH@PEG-HCuSNPs at different temperatures (A), different times (B) and different concentrations (C).


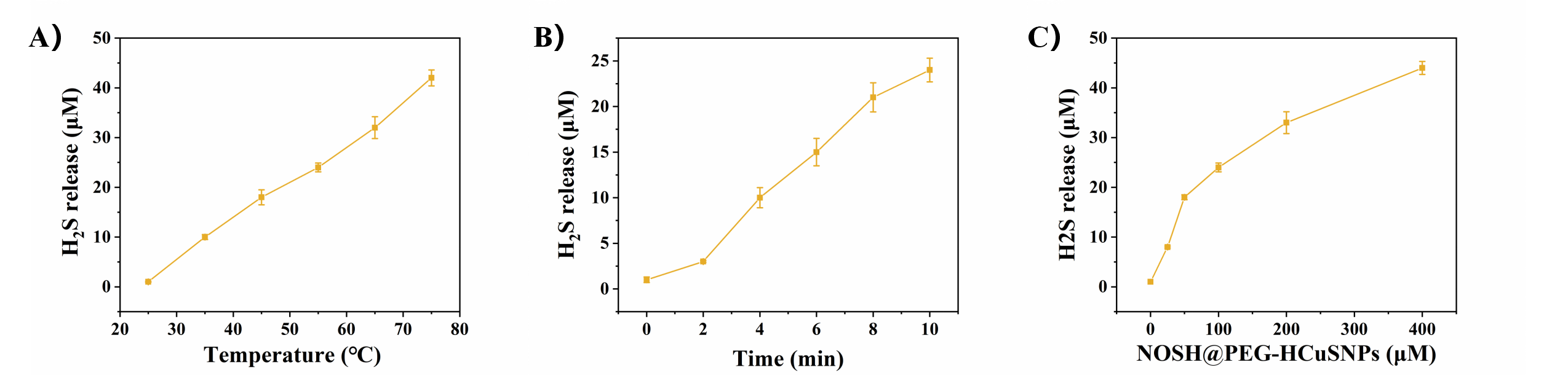


**Figure S15** Flow cytometry determination of cellular endocytosis of FITC-labeled NOSH@PEG-HCuSNPs in 4T1 cells at different treatment time points.


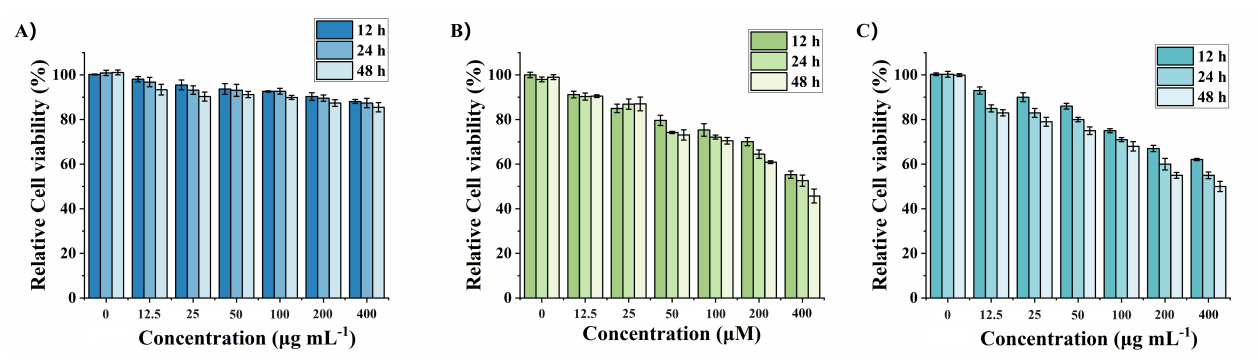


**Figure S16** A) Cell viability of 4T1 cells treated with different concentrations of NOSH@HCuSNPs (0-400 μg mL^-1^). B) Cell viability of 4T1 cells treated with different concentrations of NOSH (0-200 μM). C) Cell viability of 4T1 cells treated with different concentrations of NOSH@PEG-HCuSNPs (0-400 μg mL^-1^).


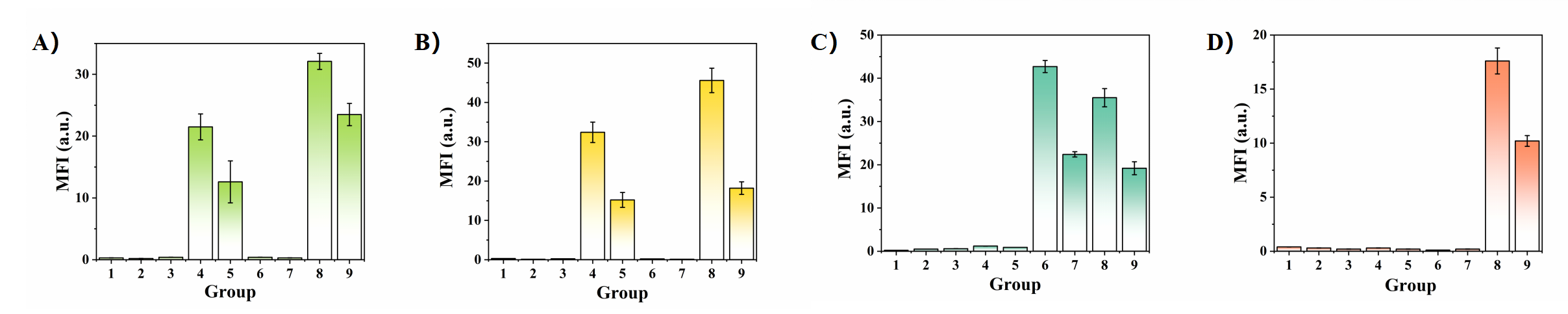


**Figure S17** Quantification of A) NO release, B) H_2_S release, C) ROS release, and D) ONOO- generation in different treatment groups.


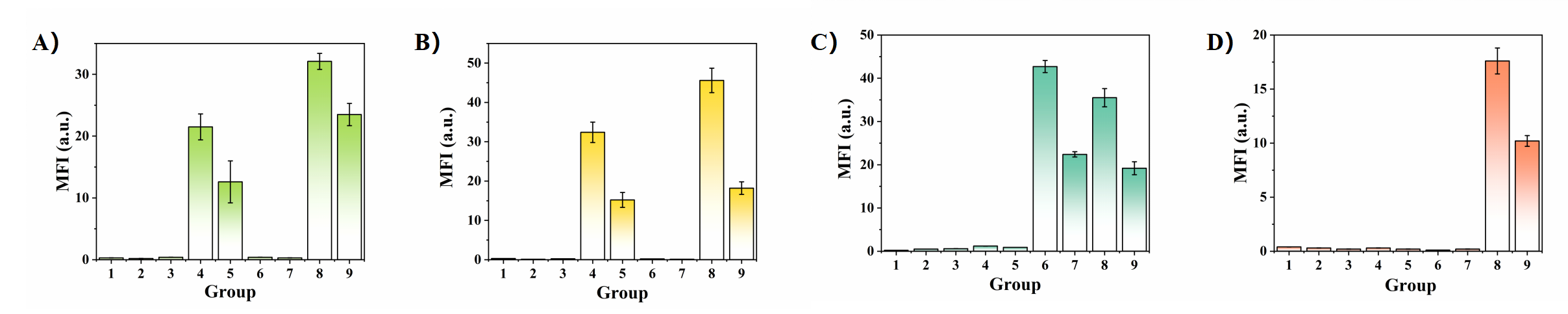


**Figure S18** Changes in intracellular ATP levels in the 9 treatment groups.





**Figure S19** The corresponding apoptosis rates of 4T1 cells after being treated with different formulas.


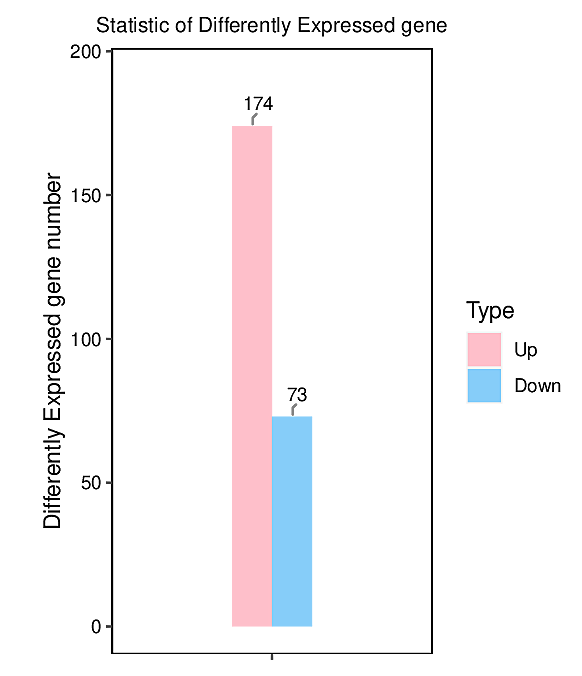


**Figure S20** The number of differentially expressed genes between the control group and the NOSH@PEG-HCuSNPs+laser treatment group.


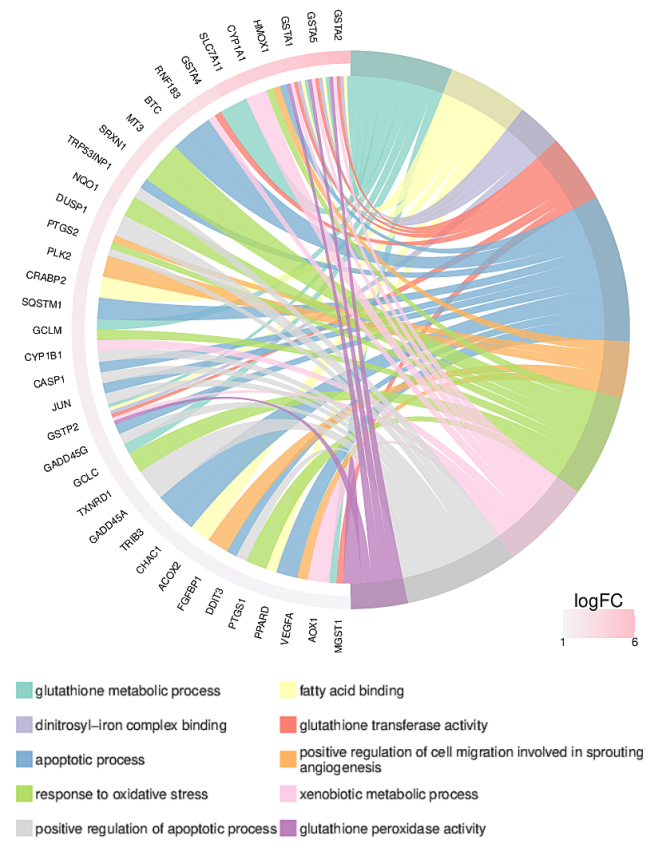


**Figure S21** GO enrichment analysis of differentially up-regulated genes showed the 10 most significantly enriched categories.

**
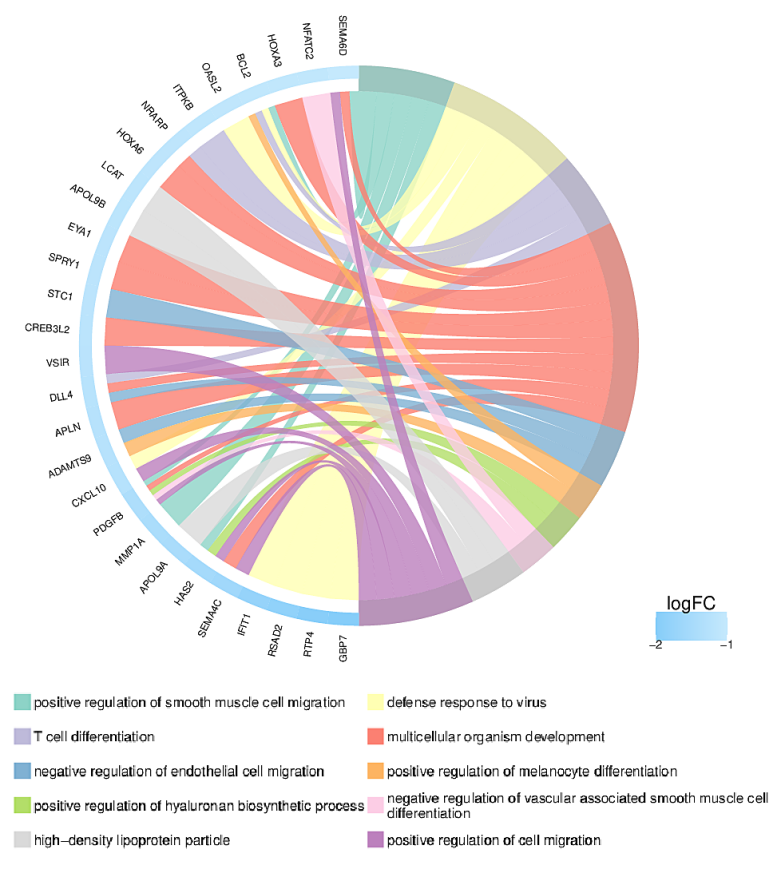
**

**Figure S22** GO enrichment analysis of differentially down-regulated genes showed the 10 most significantly enriched categories.


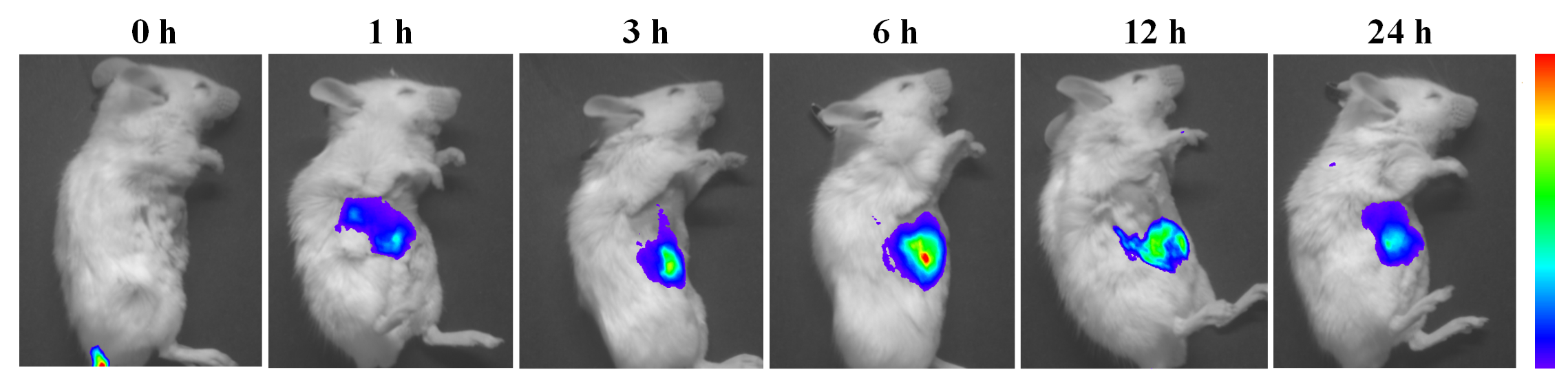


**Figure S23** Whole-body fluorescence imaging of 4T1 tumor-bearing mice intravenously injected with ICG-labeled NOSH@PEG-HCuSNPs.


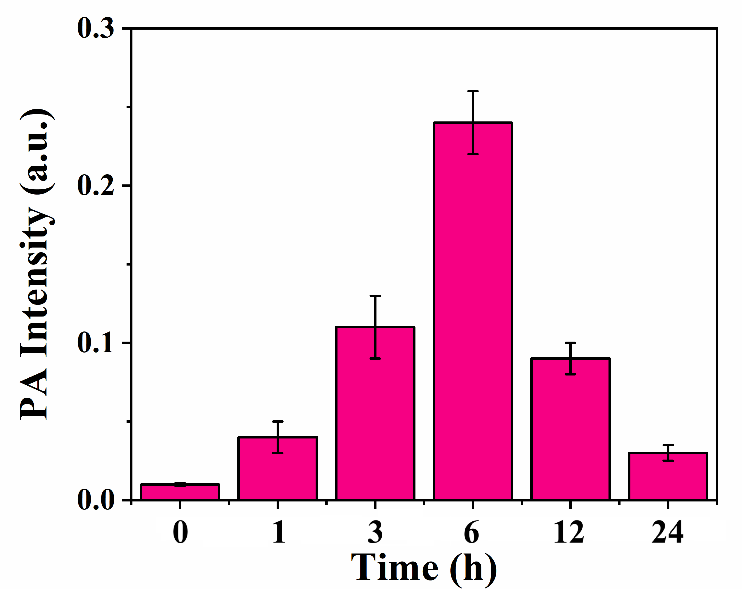


**Figure S24** PA signal intensities at tumor regions after varied treatment durations.





**Figure S25** Body weight changes of mice in each group during the treatment of single tumor model.


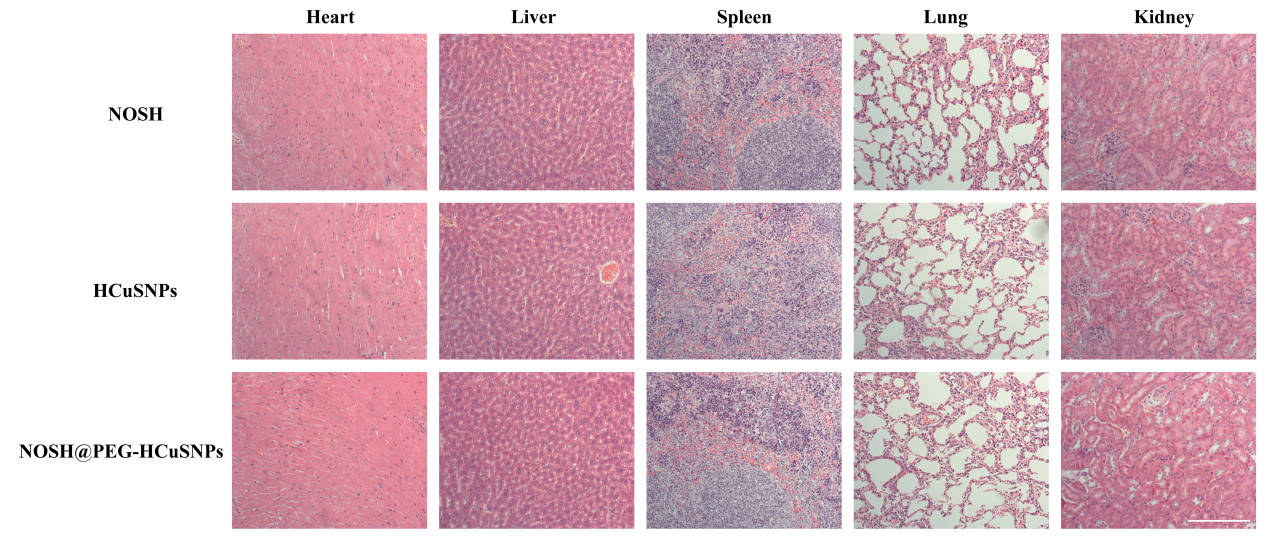


**Figure S26** H&E staining images of various organs (heart, liver, spleen, lung, and kidney) of BALB/c mice 14 days after intravenous injection of NOSH, NOSH@HCuSNPs, and NOSH@PEG-HCuSNPs.


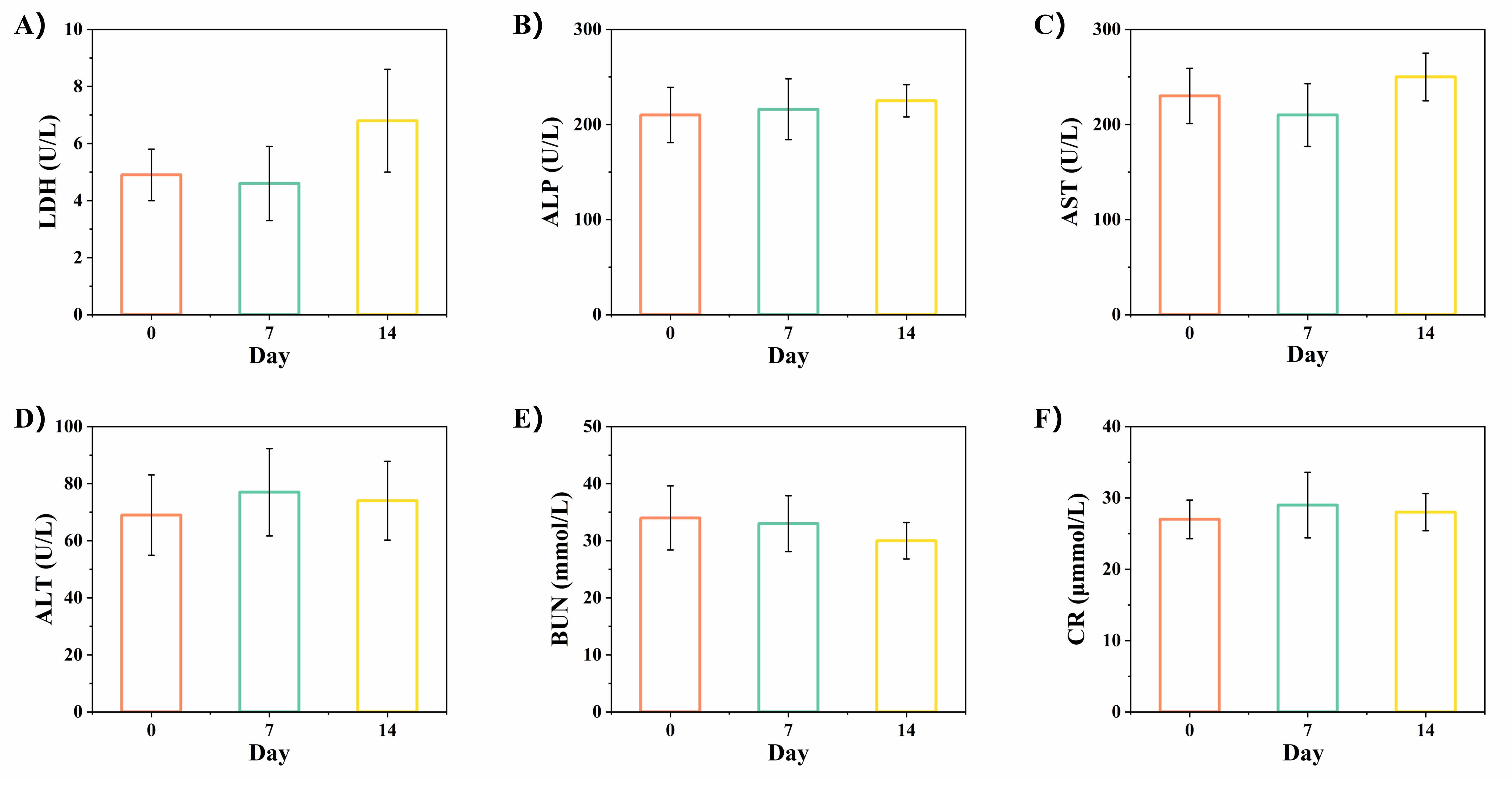


**Figure S27** Blood biochemical indices of BALB/c mice were obtained 0, 7, and 14 days after intravenous injection of NOSH@PEG-HCuSNPs (20 mg kg^-1^). Lactate dehydrogenase 1 (LDH1), transaminase (ALT), aspartate transaminase (AST), alkaline phosphatase (ALP), blood urea nitrogen (BUN), and creatinine (CR). The above data are expressed as mean and standard deviation (n = 4).


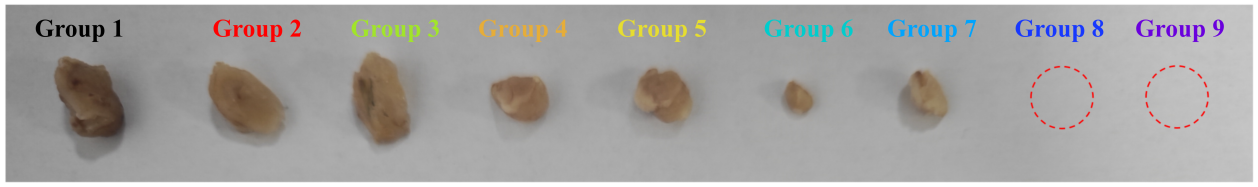


**Figure S28** Photos of tumor tissues from each group removed after different treatments.


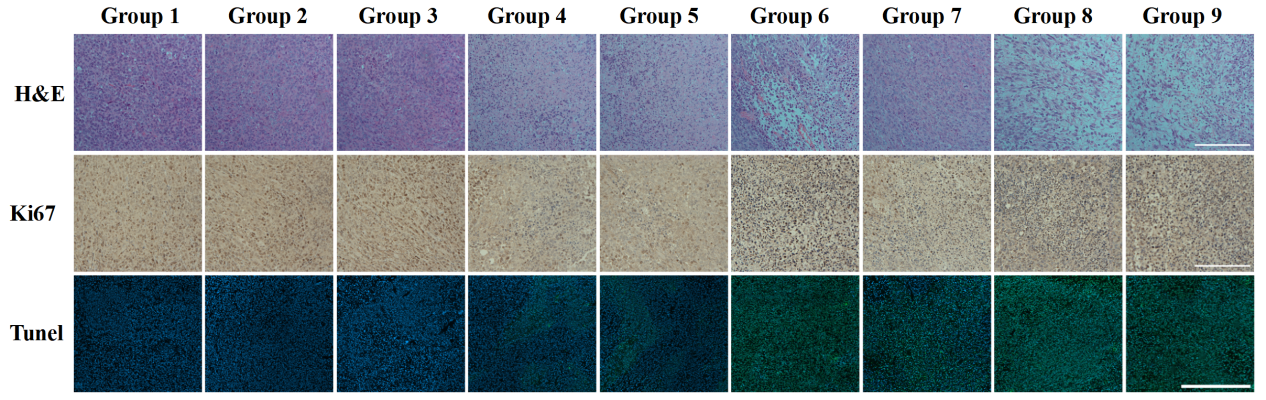


**Figure S29** H&E staining, Tunel staining, and Ki67 staining images of tumor tissue sections of each group after different treatments.


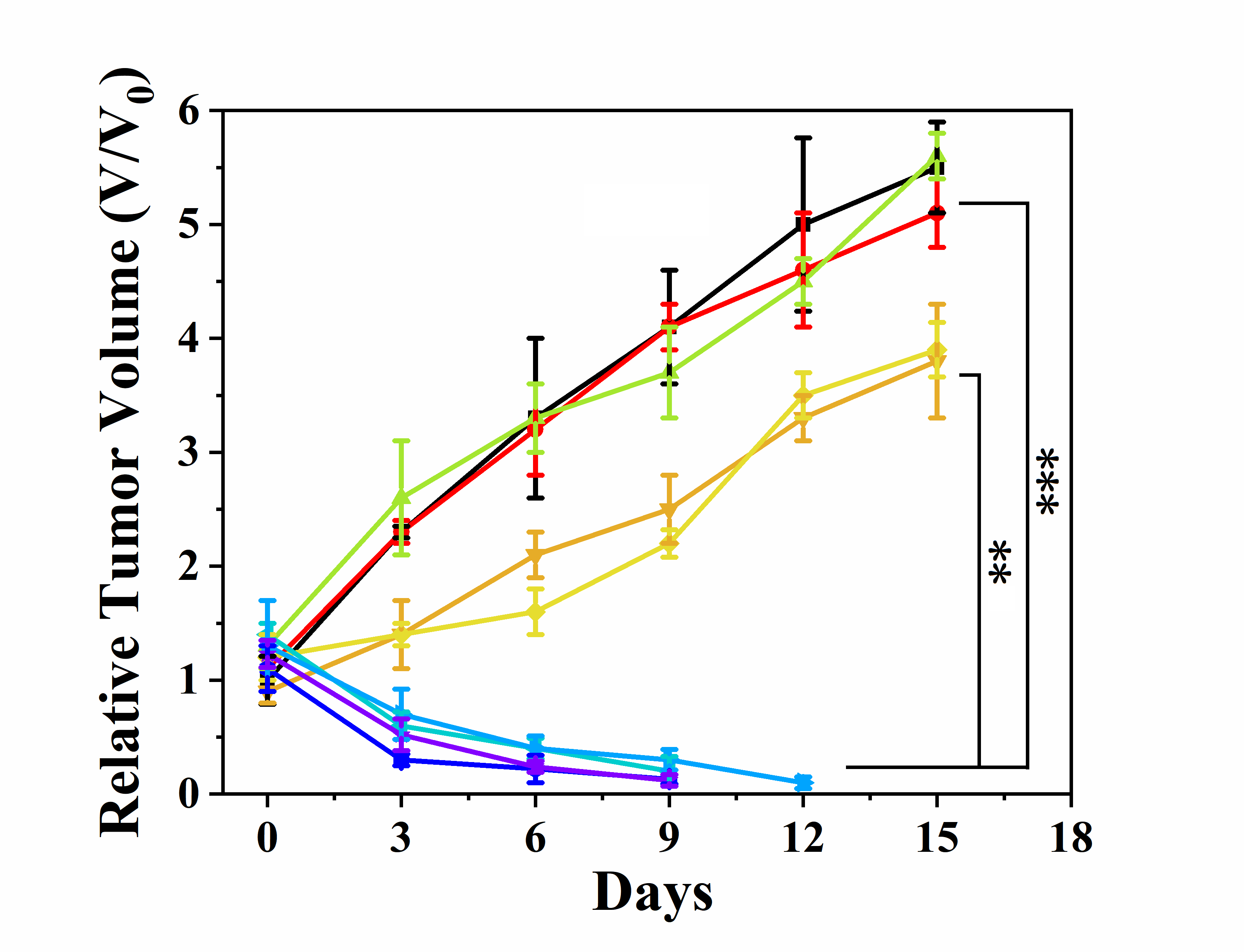


**Figure S30** The curve of primary tumor volume changes in the distal tumor model of each group after different treatments.


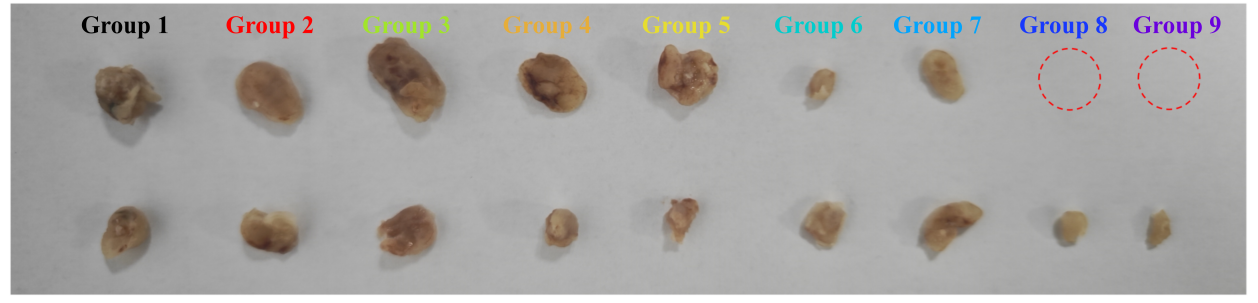


**Figure S31** Photographs of distal tumor tissues of each group removed after different treatments.





**Figure S32** Body weight changes of mice in each group during the treatment of distal model.
